# 3D cine-magnetic resonance imaging using spatial and temporal implicit neural representation learning (STINR-MR)

**DOI:** 10.1088/1361-6560/ad33b7

**Published:** 2024-04-15

**Authors:** Hua-Chieh Shao, Tielige Mengke, Jie Deng, You Zhang

**Affiliations:** 1 The Medical Artificial Intelligence and Automation (MAIA) Laboratory, Department of Radiation Oncology, University of Texas Southwestern Medical Center, Dallas, TX 75390, United States of America

**Keywords:** 3D cine-MRI reconstruction, dynamic motion, implicit neural representation, multiresolution hash encoding

## Abstract

*Objective*. 3D cine-magnetic resonance imaging (cine-MRI) can capture images of the human body volume with high spatial and temporal resolutions to study anatomical dynamics. However, the reconstruction of 3D cine-MRI is challenged by highly under-sampled k-space data in each dynamic (cine) frame, due to the slow speed of MR signal acquisition. We proposed a machine learning-based framework, spatial and temporal implicit neural representation learning (STINR-MR), for accurate 3D cine-MRI reconstruction from highly under-sampled data. *Approach*. STINR-MR used a joint reconstruction and deformable registration approach to achieve a high acceleration factor for cine volumetric imaging. It addressed the ill-posed spatiotemporal reconstruction problem by solving a reference-frame 3D MR image and a corresponding motion model that deforms the reference frame to each cine frame. The reference-frame 3D MR image was reconstructed as a spatial implicit neural representation (INR) network, which learns the mapping from input 3D spatial coordinates to corresponding MR values. The dynamic motion model was constructed via a temporal INR, as well as basis deformation vector fields (DVFs) extracted from prior/onboard 4D-MRIs using principal component analysis. The learned temporal INR encodes input time points and outputs corresponding weighting factors to combine the basis DVFs into time-resolved motion fields that represent cine-frame-specific dynamics. STINR-MR was evaluated using MR data simulated from the 4D extended cardiac-torso (XCAT) digital phantom, as well as two MR datasets acquired clinically from human subjects. Its reconstruction accuracy was also compared with that of the model-based non-rigid motion estimation method (MR-MOTUS) and a deep learning-based method (TEMPEST). *Main results*. STINR-MR can reconstruct 3D cine-MR images with high temporal (<100 ms) and spatial (3 mm) resolutions. Compared with MR-MOTUS and TEMPEST, STINR-MR consistently reconstructed images with better image quality and fewer artifacts and achieved superior tumor localization accuracy via the solved dynamic DVFs. For the XCAT study, STINR reconstructed the tumors to a mean ± SD center-of-mass error of 0.9 ± 0.4 mm, compared to 3.4 ± 1.0 mm of the MR-MOTUS method. The high-frame-rate reconstruction capability of STINR-MR allows different irregular motion patterns to be accurately captured. *Significance*. STINR-MR provides a lightweight and efficient framework for accurate 3D cine-MRI reconstruction. It is a ‘one-shot’ method that does not require external data for pre-training, allowing it to avoid generalizability issues typically encountered in deep learning-based methods.

## 1. Introduction

Magnetic resonance imaging (MRI) is a non-invasive modality that can capture morphological and functional characteristics to detect and diagnose health problems (Constantine *et al* 2004, Bartsch *et al* 2006, Frisoni *et al* 2010, Dregely *et al* 2018), and to provide image guidance for treatment planning and interventions (Cleary and Peters 2010, Pollard *et al* 2017, Stemkens *et al* 2018), without utilizing ionizing radiation. With advances in hardware designs and innovations in pulse sequences for efficient k-space sampling, time-resolved cine-MRI can now be acquired to visualize time-varying dynamic processes (Nayak *et al* 2022), including cardiac motion (Rajiah *et al* 2023), blood perfusion (Jahng *et al* 2014), speech and vocal production (Lingala *et al* 2016), and gas flow in lungs (Wild *et al* 2003), etc. However, due to the still-limited speed of k-space data sampling, many of the current cine-MRI applications are limited to 2D, although 3D cine-MRI is highly desired to capture the complex motion/deformation of anatomical volumes (Seppenwoolde, Shirato *et al* 2002) to guide diagnosis or treatments, such as the MR-guided radiotherapy (Corradini *et al* 2019, Hall *et al* 2019, Witt *et al* 2020). Considering a pulse sequence with a 4 ms repetition time (TR), for a 100 ms temporal resolution there are only 25 available k-space readout lines (i.e. frequency encoding) to reconstruct a 3D cine-MRI, which is extremely under-sampled. Recently, 4D-MRI was developed (Stemkens *et al* 2018, Curtis and Cheng 2022), by repeatedly measuring the dynamic processes and then retrospectively sorting the acquired MR data into ‘motion bins’ to capture a nominal, averaged motion cycle. The repeated measurements help to secure sufficient data to overcome the under-sampling challenge of the dynamic reconstruction problem. However, the repeated measurements and the subsequent motion sorting implicitly assume that the underlying anatomical motion is regular and reproducible, which usually does not reflect the clinical reality (Yasue *et al* 2022). Irregular motion patterns can result in degraded image quality (blurriness, ghosting, and other motion artifacts). Also, the averaged motion curves of 4D-MRIs cannot represent irregular motion that can be important to determine the appropriate radiotherapy margin size or to accumulate the delivered radiation dose. Therefore, reconstructing dynamic 3D cine-MRI is highly desirable in clinics, but remains a challenging problem to solve due to extreme under-sampling.

In the past decades, substantial efforts have been put into developing reconstruction algorithms for under-sampled k-space measurements. These reconstruction algorithms can be categorized into two main types: model-based iterative algorithms and learning-based techniques (Ravishankar *et al* 2020). The first type relies on parallel imaging (Hamilton *et al* 2017) and compressed sensing, as well as the corresponding system models (Lustig *et al* 2007, Feng *et al* 2017). Parallel imaging uses the spatial information from the sensitivity profiles of phased array coils to remove aliasing MRI artifacts or to recover missing k-space data. Compressed sensing regularizes the sparsity of MR images in transformed domains to aid image reconstruction using incoherent measurements. For time-resolved MRI, the spatiotemporal correlation was further exploited to balance the temporal resolution, the spatial resolution, and the image quality (Tsao *et al* 2003, Jung *et al* 2009, Uecker *et al* 2010, Asif *et al* 2013, Feng *et al* 2016). However, the acceleration factors in these model-based algorithms remain limited (typically $\lesssim $ 10), and compressed sensing-based regularization may lead to overly-smoothed, blurred images under extreme under-sampling scenarios (Jaspan *et al* 2015). Moreover, these algorithms were mostly driven by non-linear iterative optimization and thus computationally demanding, leading to lengthy reconstruction durations. Accordingly, these methods were largely limited to 2D reconstructions with small numbers of cine frames. To achieve higher acceleration factors to enable 3D cine-MRI reconstruction, deformable image registration was introduced to replace the traditional reconstruction approaches. These deformation-based algorithms reconstructed dynamic MRI frames by estimating the motion fields of underlying subjects with respect to a reference MR image, based on limited-sampled k-space data. The reference MR image was reconstructed either from a separate MR scan or from a subset of the dynamic MR acquisitions. In particular, Huttinga *et al* developed a framework, MR-MOTUS, for model-based non-rigid motion and dynamic 3D MRI estimation (Huttinga *et al* 2020, Huttinga *et al* 2021, Huttinga *et al* 2022). Via MR-MOTUS, a 250-frame 3D cine-MRI can be reconstructed with 30 readout lines per frame. However, the accuracy of pure deformation-driven techniques like MR-MOTUS is susceptible to the quality of the reference image. If a separate scan was used to acquire the reference image, the non-deformation intensity variations between the reference image and the dynamic MR acquisition would impact the deformation accuracy (Zhang *et al* 2017). If a subset of the dynamic MR acquisitions is used to reconstruct the reference image, the accuracy will instead be impacted by the aliasing artifacts (from under-sampling) and/or the motion artifacts (from intra-subset motion) of the reference image.

The second type of technique is leaning-based, particularly DL-based techniques (Liang *et al* 2020). Schlemper *et al* developed a cascaded network to unroll the reconstruction problem into joint reconstruction and DL-based de-aliasing (Schlemper *et al* 2018). To facilitate the learning of spatiotemporal features for dynamic reconstruction, they introduced data-sharing layers and demonstrated an 11-fold acceleration for 2D dynamic cardiac MRI. Biswas *et al* introduced a DL framework that incorporated prior information for image denoising, including patient-specific smoothness regularization on a manifold prior and a learned deep prior (Biswas *et al* 2019). The algorithm can reconstruct a 200-frame 2D cardiac MRI with 10 readout lines per frame. Huang *et al* proposed a motion-guided network comprised of three sub-networks for initial image reconstruction, motion estimation, and motion compensation (Huang *et al* 2021), which showed an 8-fold acceleration for 2D cardiac MRI. Although these DL-based methods demonstrated impressive results in cine-MRI reconstruction, the majority of these studies focused on 2D reconstructions as 3D cine-MRI reconstruction is challenged by more extreme under-sampling. Similar to the scenario of the first-type algorithms, the deformation-based approaches were also introduced into DL-based frameworks, which can potentially achieve real-time 3D cine-MRI with a high acceleration factor and low inference latency (Terpstra *et al* 2021, Shao *et al* 2022). However, similar to the deformation-driven algorithms like MR-MOTUS, the DL-based algorithms are impacted by the non-deformation intensity variations between the reference image and the dynamic MR acquisition, or the aliasing/motion artifacts of the reference image. Another major drawback of these DL-based techniques is the model uncertainty and the lack of robustness. The DL-based techniques need to be partially or fully pre-trained, and any data distribution shifts between training and testing can lead to generalizability issues and substantially degrade their accuracy (Zech *et al* 2018, Kelly *et al* 2019, Full *et al* 2021).

In addition to the above DL-based methods, recently a new machine learning technique, implicit neural representation (INR), has found potential applications in medical image reconstruction, registration, and analysis (Khan and Fang 2022, Molaei *et al* 2023, Rao *et al* 2023). INR uses neural networks to implicitly represent the physical features of objects (e.g. geometry and material properties such as opacity, x-ray attenuation coefficient, or MR intensity) in a complex 3D scene (Mildenhall *et al* 2022, Tewari *et al* 2022). A neural network in INR functions as a universal function approximator (Hornik *et al* 1989) which takes spatial coordinates of a scene (MR image voxel coordinates, for instance) as inputs and continuously maps them to the desired physical features (MR intensities at the queried voxels) via the learning process. The implicit representation via networks allows the underlying MR image to be captured compactly without specifying the function form in advance (Tewari *et al* 2022), and allows natural super-resolution since the MR image intensity can be queried at arbitrary, non-integer coordinates (Chen *et al* 2022). In contrast to DL-based methods, which typically require a large dataset for pre-training, INR can be trained in a single shot by directly using limited samples of the studied subject to optimize the network parameters. Therefore, INR is learning efficient and can avoid the generalizability issues typically encountered in DL-based techniques. With these advantages, INR has been applied to solve x-ray-based and MR-based reconstruction problems from sparse-view measurements (Shen *et al* 2022, Zha *et al* 2022). Furthermore, INR-based reconstruction algorithms for dynamic computed tomography (CT) and cone-beam CT were also developed (Reed *et al* 2021, Zhang *et al* 2023).

Inspired by our recent work in INR-based cone-beam CT reconstruction (Zhang *et al* 2023), in this work we proposed a joint reconstruction and deformable registration-based framework using spatial and temporal INRs for dynamic 3D cine-MRI reconstruction (STINR-MR). STINR-MR uses spatiotemporal INRs to learn, reconstruct, and map 3D cine-MRI volumes and the corresponding time-varying motion. It reconstructs a reference-frame image and solves time-varying motion fields with respect to the reference frame to derive corresponding 3D cine-MR images. Compared with pure deformation-driven methods like MR-MOTUS, STINR allows simultaneous reconstruction and motion modeling to solve/optimize the reference-frame image directly and iteratively from the cine k-space data, and thus is less affected by the non-deformation variations between the reference MR image and the cine-MR images. The reconstruction/optimization of the reference MR image using the full cine k-space data also renders it less susceptible to the aliasing/under-sampling artifacts. In contrast to our prior STINR work, we used a powerful learning-based input encoding scheme (multi-resolution Hash encoding) for STINR-MR, rendering it a light-weight and efficient framework capable of reconstructing 3D cine-MRIs of >1000 frames within a short duration ($\lesssim $20 min). STINR-MR was evaluated by MR data simulated from a 4D extended cardiac-torso (XCAT) digital phantom (Segars *et al* 2010) featuring various regular/irregular breathing patterns. It was also evaluated by an MRI dataset of five patients from our institute and an MRI dataset of a healthy human subject from a publicly available repository (Huttinga *et al* 2021). The reconstruction and motion tracking accuracy of STINR-MR was also compared with that of MR-MOTUS and a DL-based method TEMPEST (Terpstra *et al* 2021).

## 2. Materials and methods

### 2.1. Problem formulation

Let ${\left\{{{\boldsymbol{w}}}_{t}({\boldsymbol{k}})\right\}}_{t=0}^{{N}_{t}-1}$ be a series of consecutive 3D MR acquisitions in k-space, where ${{\boldsymbol{w}}}_{t}({\boldsymbol{k}})$ denotes the acquired MR signals at coordinates ***k*** and is labeled by the frame index *t*, and ${N}_{t}$ denotes the total number of acquired frames. A frame here refers to a cine-MR volume of a sufficient temporal resolution in the time series, so that the dynamic process under study can be considered quasi-static for each frame. In this study, we were interested in respiration-induced motion, which is a major source of uncertainties in radiotherapy (Stemkens *et al* 2018). Dynamic cine-MRI reconstruction aims to generate the moving sequence of the underlying subjects ${\left\{{{\boldsymbol{z}}}_{t}({\boldsymbol{x}})\right\}}_{t=0}^{{N}_{t}-1}$ in the image domain (i.e. time-varying cine-MR images), which are matched to the acquired signals in k-space ${\left\{{{\boldsymbol{w}}}_{t}({\boldsymbol{k}})\right\}}_{t=0}^{{N}_{t}-1}$(Fessler 2010, Hansen and Kellman 2015). Here, ${\boldsymbol{x}}$ denotes the voxel coordinates of the reconstructed images. The reconstruction is formulated as an optimization problem with a regularization term:

\begin{eqnarray*}\left\{{\hat{{\boldsymbol{z}}}}_{t}\right\}=\mathop{{\mathrm{argmin}}}\limits_{\left\{{{\boldsymbol{z}}}_{t}\right\}}\,\left({\unicode{x02016}F\left\{{{\boldsymbol{z}}}_{t}({\boldsymbol{x}})\right\}-\left\{{{\boldsymbol{w}}}_{t}({\boldsymbol{k}})\right\}\unicode{x02016}}^{2}+\lambda \,R\left(\left\{{{\boldsymbol{z}}}_{t}({\boldsymbol{x}})\right\}\right)\right),\end{eqnarray*}

where *F* is an operator combining the coil sensitivity map and the Fourier transform matrix corresponding to the k-space sampling pattern. *R* is the regularization term weighted by the factor $\lambda .$ The data fidelity term (first term) of equation (1) enforces the data consistency between the reconstructed images $\left\{{\hat{{\boldsymbol{z}}}}_{t}\right\}$ and the k-space MR acquisitions $\left\{{{\boldsymbol{w}}}_{t}({\boldsymbol{k}})\right\}.$ The regularization term introduces prior knowledge of the images under study (i.e. sparsity in transformed domains) to facilitate the reconstruction and prevent overfitting in the optimization process.

To overcome the k-space under-sampling issue, STINR-MR adopted a joint reconstruction and deformable registration approach, viewing each frame of the cine-MR images as a deformed version of a reference-frame image ${{\boldsymbol{z}}}_{\mathrm{ref}}\left({\boldsymbol{x}}\right):$

\begin{eqnarray*}{{\boldsymbol{z}}}_{t}\left({\boldsymbol{x}}\right)={{\boldsymbol{z}}}_{\mathrm{ref}}\left({\boldsymbol{x}}+{{\boldsymbol{d}}}_{t}\left({\boldsymbol{x}}\right)\right),\end{eqnarray*}

where ${{\boldsymbol{d}}}_{t}\left({\boldsymbol{x}}\right)$ is the deformation vector field (DVF) at the cine frame *t*. Equation (2) assumes the existence of a reference frame and that the intra-scan motion can be described by DVFs, which is supported by the fact that for images acquired within a single scan, the MR intensities are considered stable and the major variations are caused by anatomical motion. Note that the assumption of equation (2) excludes short-term physiological phenomena that may significantly change the MR intensities [e.g. contrast agents in dynamic contrast-enhanced MRI (Sourbron and Buckley 2013, Petralia *et al* 2020)], which is not considered in this study. The reference-frame image ${{\boldsymbol{z}}}_{\mathrm{ref}}\left({\boldsymbol{x}}\right)$ serves as a template from which all cine-MR images are derived, and itself may not necessarily correspond to an exact frame in the sequence $\left\{{{\boldsymbol{z}}}_{t}\right\}.$ Via equations (1) and (2), STINR-MR decoupled the ill-posed spatiotemporal reconstruction problem into reconstructing a reference MR image ${{\boldsymbol{z}}}_{\mathrm{ref}}\left({\boldsymbol{x}}\right)$ and solving the corresponding dynamic motion $\left\{{{\boldsymbol{d}}}_{t}\left({\boldsymbol{x}}\right)\right\},$ thus reducing the complexity of the reconstruction. In the following subsections, we first overviewed the workflow of STINR-MR, followed by details of the network architecture and training scheme. Afterward, the dataset and evaluation schemes were presented.

### 2.2. STINR-MR workflow overview

**Figure 1.**
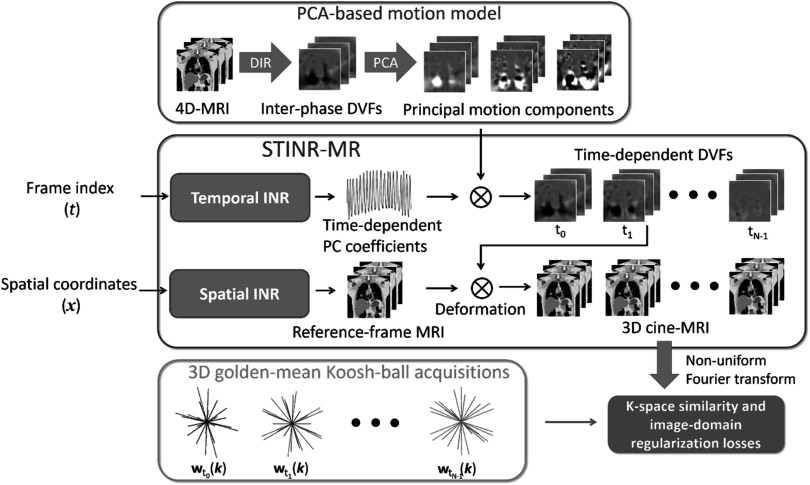
Overview of the workflow of 3D cine-MRI reconstruction (STINR-MR). The 3D cine-MRI reconstruction was based on a joint reconstruction and deformable registration approach by which each frame of the 3D cine-MRI was generated by deforming a reference-frame image (equation (2)). STINR-MR utilized a spatial implicit neural representation (INR) to reconstruct the reference-frame image, and a temporal INR to represent time-dependent motion. A principal component analysis (PCA)-based patient-specific motion model was incorporated into the framework to regularize the motion. The k-space readout was based on the 3D golden-mean Koosh-ball trajectory. The network training was driven by a k-space similarity loss and an image-domain regularization loss such that the k-space data of each reconstructed image matched with the corresponding measured k-space signals. DIR: deformable image registration. PCA: principal component analysis. DVF: deformation vector field. INR: implicit neural representation.

Figure 1 illustrates the workflow of STINR-MR. STINR-MR consisted of a spatial INR and a temporal INR (middle box of figure 1). The spatial INR represents the reference-frame MR image and the temporal INR represents the intra-scan dynamic motion. Combining both INRs, 3D cine-MRI can be derived to represent spatiotemporal dynamics. In detail, the input into the spatial INR was a voxel coordinate ***x***, and the output was the MRI value at the queried coordinate (i.e. ${{\boldsymbol{z}}}_{\mathrm{ref}}\left({\boldsymbol{x}}\right)$). The entire volume of the reference frame can then be generated by querying all voxel coordinates within the region of interest. For the intra-scan motion (DVFs), the dimensionality of the solution space is extremely large, involving $\geqslant $10^8^ degrees of freedom (Huttinga *et al* 2021). To regularize the solution of motion, we incorporated a principal component analysis (PCA)-based patient-specific motion model into the framework (top box of figure 1). PCA-based motion model introduced prior motion modes to significantly reduce the dimensionality of the unknown DVFs (Zhang *et al* 2013, Zhang *et al* 2019, Zhang *et al* 2023). To derive the PCA-based motion model, a previously acquired, motion-binned 4D-MRI can be used. Alternatively, the motion-binned 4D-MRI can also be directly derived from the cine-MRI acquisition. We obtained the inter-phase DVFs by registering the motion-binned 4D-MR images to the end-of-exhale bin, which is relatively stable with fewer artifacts (Vedam *et al* 2001, Heerkens *et al* 2014, Lever *et al* 2014). The *principal motion components* can then be solved by performing PCA on the inter-phase DVFs of the 4D-MRI. The principal motion components can be considered as a basis set $\left\{{{\boldsymbol{e}}}_{i}({\boldsymbol{x}})\right\}$ spanning a Hilbert space and maximally accounting for the motion variance in the inter-phase DVFs $\left\{{{\boldsymbol{D}}}_{p}({\boldsymbol{x}})\right\}:$

\begin{eqnarray*}{\left\{{{\boldsymbol{e}}}_{i}\left({\boldsymbol{x}}\right)\right\}}_{i=1}^{{N}_{{pc}}}=\mathop{\mathrm{argmax}}\limits_{\left\{{{\boldsymbol{e}}}_{i}\right\}}\left\{\mathrm{var}\left[\displaystyle \sum _{p=1}^{{N}_{{bin}}}{{\boldsymbol{D}}}_{p}\left({\boldsymbol{x}}\right){\mathrm{\cdot }}{{\boldsymbol{e}}}_{i}\left({\boldsymbol{x}}\right)\right]\right\}\end{eqnarray*}

${\mathrm{such\; that}}$

\begin{eqnarray*}\mathrm{cov}\left[\displaystyle \sum _{p=1}^{{N}_{{bin}}}{{\boldsymbol{D}}}_{p}\left({\boldsymbol{x}}\right){\mathrm{\cdot }}{{\boldsymbol{e}}}_{i}\left({\boldsymbol{x}}\right),\displaystyle \sum _{p=1}^{{N}_{{bin}}}{{\boldsymbol{D}}}_{p}\left({\boldsymbol{x}}\right){\mathrm{\cdot }}{{\boldsymbol{e}}}_{j}\left({\boldsymbol{x}}\right)\right]=0\,{\mathrm{for}}\,i\ne j\,{\mathrm{and}}\,{{\boldsymbol{e}}}_{i}\left({\boldsymbol{x}}\right){\mathrm{\cdot }}{{\boldsymbol{e}}}_{j}\left({\boldsymbol{x}}\right)={\delta }_{{ij}}\end{eqnarray*}

where ${N}_{{pc}}$ denotes the dimensionality of the space spanned by $\left\{{{\boldsymbol{e}}}_{i}({\boldsymbol{x}})\right\},$ ${N}_{{bin}}$ denotes the number of the motion bins of the 4D-MRI, and $\mathrm{var}$ and $\mathrm{cov}$ respectively denote the variance and covariance of their arguments. ${\delta }_{{ij}}$ denotes the Kronecker delta, and the inner product is defined in the Hilbert space of the motion fields. An arbitrary respiratory DVF can be represented as a linear combination of these principal motion components. Here, we used the first three principal motion components (together with the mean inter-phase DVF) as the basis, as the first three components were shown sufficient to accurately describe the respiratory motion (Li *et al* 2011). Through this strategy, the PCA-based motion model reduced the dimensionality of the unknown DVFs from $\geqslant $ 10^8^ to 9. With the PCA-based motion model, we used a temporal INR to represent the PCA weightings, in the form of nine PC coefficients (i.e. three principal motion components × three Cartesian directions) at each queried frame index. The principal motion components, scaled by the weighting outputs from the temporal INR, were superposed to generate frame-specific DVFs:

\begin{eqnarray*}{{\boldsymbol{d}}}_{t}\left({\boldsymbol{x}}\right)={{\boldsymbol{e}}}_{0}\left({\boldsymbol{x}}\right)+\displaystyle \sum _{i=1}^{3}{w}_{i}\left(t\right)\times {{\boldsymbol{e}}}_{i}\left({\boldsymbol{x}}\right),\end{eqnarray*}

where ${{\boldsymbol{e}}}_{0}\left({\boldsymbol{x}}\right)$ is the mean DVF of $\left\{{{\boldsymbol{D}}}_{p}({\boldsymbol{x}})\right\},$ ${{\boldsymbol{e}}}_{i}\left({\boldsymbol{x}}\right)$ is the *i*th principal motion component, and ${w}_{i}\left(t\right)$ is the corresponding PC weighting. The time sequences of the PC weightings, output via the temporal INR, form the time series of the motion fields $\left\{{{\boldsymbol{d}}}_{t}\left({\boldsymbol{x}}\right)\right\}$ to capture the dynamic motion. Finally, the 3D cine-MR images were reconstructed by applying the sequence of $\left\{{{\boldsymbol{d}}}_{t}\left({\boldsymbol{x}}\right)\right\}$ to the reference-frame MRI, as in equation (2).

As shown in the workflow, the spatial INR and the temporal INR were jointly solved (trained), by matching the projected k-space data of reconstructed 3D cine-MR images to the acquired k-space data. The training was purely driven by the acquired data of each dynamic MR acquisition in an iterative fashion, thus allowing ‘one-shot’ learning. The joint training scheme allowed concurrent update and refinement of the reference frame and the intra-scan motion via all k-space data, thus improving the overall accuracy and consistency throughout the time series.

### 2.3. Network architectures and the training scheme

#### 2.3.1. The spatial implicit neural representation

**Figure 2.**
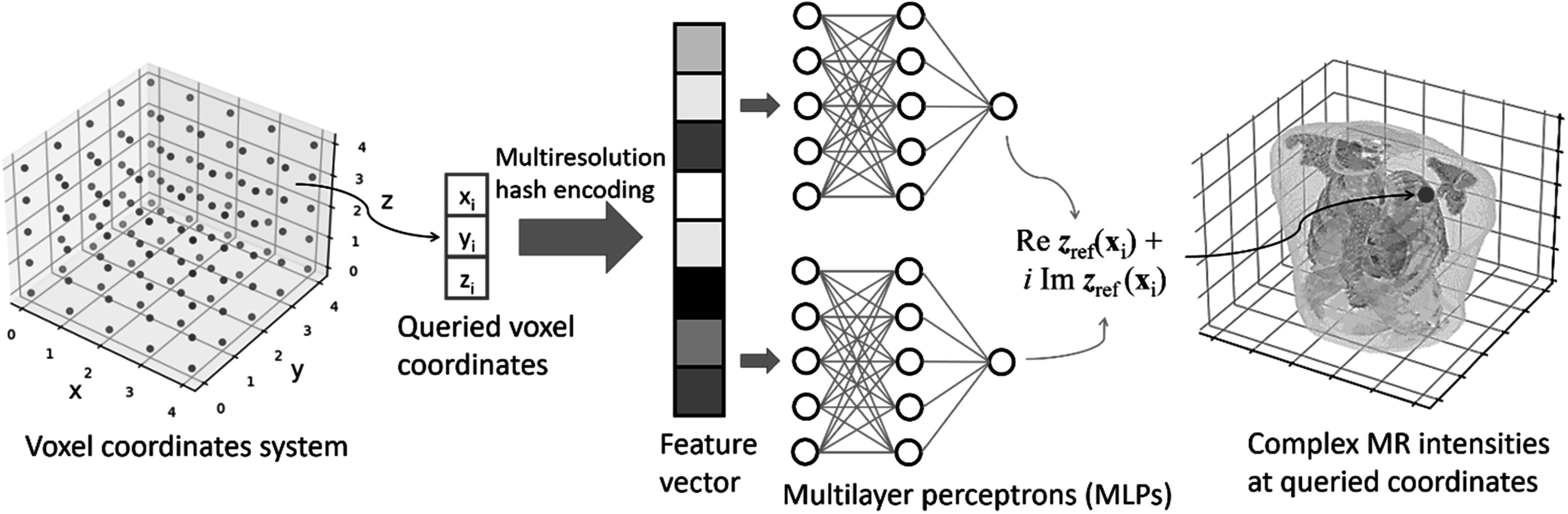
Workflow and network architecture of the spatial INR. The spatial INR network took a voxel coordinate *x* as the input, and output the complex-valued MR intensity at the queried voxel. The input coordinate was first mapped to a higher dimension space by a multiresolution hash encoding scheme, and the resulting feature vector was input into a subsequent structure of multilayer perceptrons (MLPs). Two independent MLPs were respectively used to represent the real and imaginary parts of the image. The volume reconstruction was achieved by querying all voxel coordinates within the region of interest.

**Figure 3.**
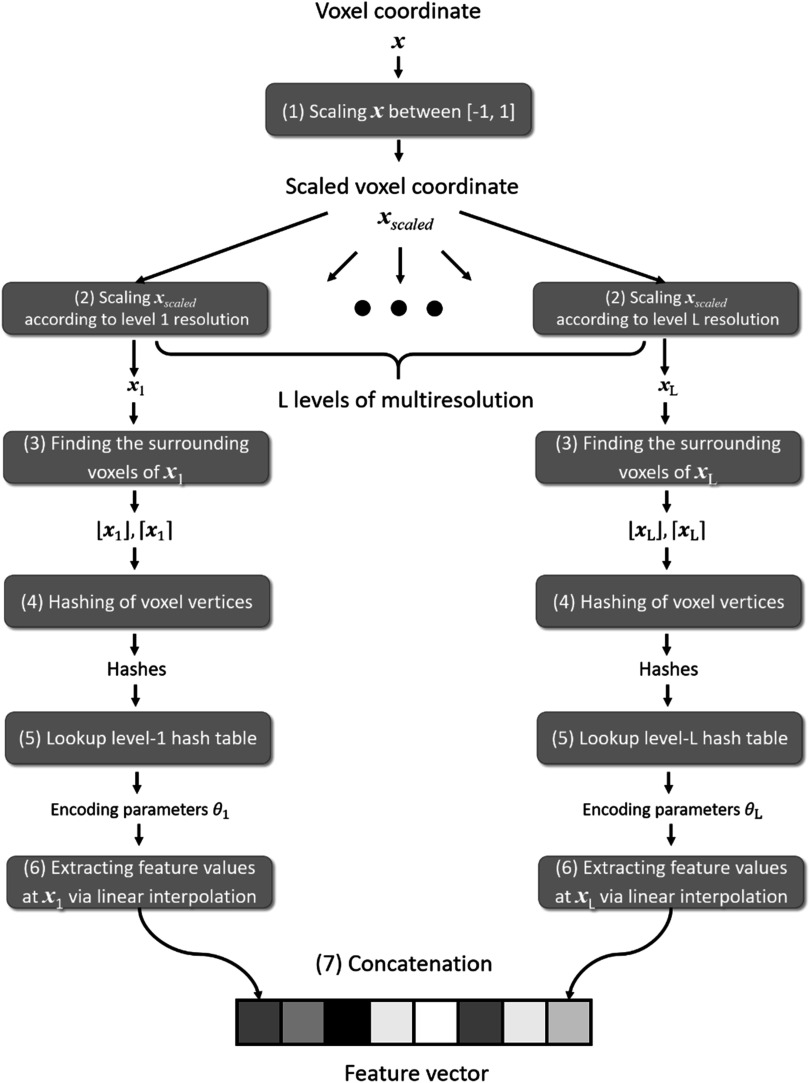
Workflow of the multiresolution hash encoding (Muller *et al* 2022). The multiresolution hash encoding mapped a voxel coordinate to a feature vector in a higher dimension space via learnable hash tables. The encoding scheme was through a multiresolution approach that progressively increases the spatial resolution at higher levels. At each level, the encoding scheme sets up a grid of vertices with integer indexes based on the resolution of the level. Then the encoding scheme first mapped the input voxel coordinate ***x*** to this grid system by scaling the coordinate ***x*** in steps (1) and (2). Through steps (3)–(5), a hash function mapped the indexes of the surrounding vertices of the scaled coordinate to the learnable hash table to retrieve the encoding parameters. The feature values of the voxel coordinate were subsequently extracted based on the relative position of the voxel to its surrounding vertices in step (6), via linear interpolations of the encoding parameters. Finally, the extracted feature values of all levels were concatenated in step (7).

Figure 2 illustrates the workflow of the reference-frame MRI reconstruction. As mentioned in section 2.2, the spatial INR mapped 3D voxel coordinates to the corresponding complex-valued MR intensities. The INR was constructed via multilayer perceptrons (MLPs). As the MLPs have shown difficulties in learning high-frequency image features directly (Tancik *et al* 2020), the input coordinates need to be pre-processed by a learning-based position encoding scheme before inputting into the MLPs, to promote the learning of high-frequency features. We used the multiresolution hash encoding (Muller *et al* 2022), which mapped the 3D space to a higher dimension space, using a spatial hash function and a multiresolution hierarchy of hash tables (figure 3). The hash tables were learning-based with trainable parameters, allowing efficient and adaptive encoding. The output of the hash encoding was a feature vector whose length depended on the number of multiresolution levels. Multiresolution hash encoding has shown advantages over other encoding schemes in terms of the representation quality, the versatility of usage, and the training speed (Muller *et al* 2022). In addition, by the multiresolution hash encoding, the depth of the MLPs can be reduced, allowing smaller and more efficient architectures to be deployed. Therefore, the training time can be significantly shortened. For the multiresolution hash encoding, we used hyper-parameter values recommended by the literature (Muller *et al* 2022), and they were summarized in table 1. The range of the voxel coordinate system was scaled between −1 and 1 prior to the hash encoding.

**Table 1.** Hyper-parameters of the multiresolution hash encoding.

Hyper-parameter	Value
Number of levels	16
Maximum entries per level	2^19^
Number of feature dimensions per entry	2
Coarsest resolution	16
Finest resolution	10509

Since MR images are complex-valued, two independent MLPs were used for the spatial INR to represent the real and imaginary parts of the image, respectively. Each MLP comprised an input, a hidden, and an output layer, whose feature numbers were 32, 32, and 1, respectively. Given that the real and imaginary parts of the MR value correspond to the same anatomical structure and geometrical location, the same hash-encoded feature vector was shared by the two MLPs. We used the same periodic activation function as a previous study (Sitzmann *et al* 2020), and initialized the learnable parameters of the MLPs in a similar way.

#### 2.3.2. The temporal implicit neural representation

**Figure 4.**
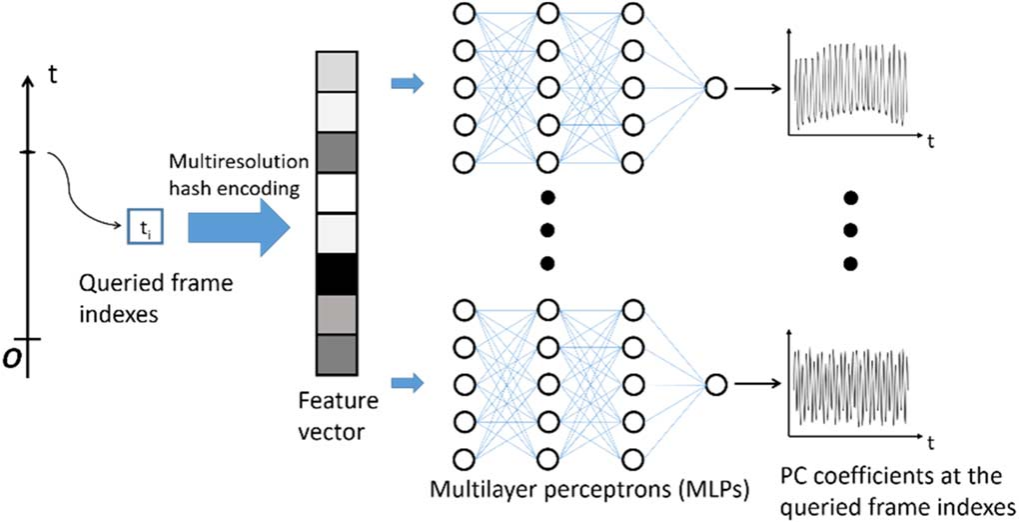
Workflow and network architecture of the temporal INR. The temporal INR took as input a frame index of the MR acquisition and output principal component (PC) weightings/coefficients at the queried frame index. Similar to the spatial INR (figure 2), the input coordinates were first mapped to a higher dimension space by the multiresolution hash encoding. Nine parallel MLPs were used to map the encoded feature vector to nine frame-dependent PC coefficients. The complete temporal sequence of PC coefficients was obtained by querying all frame indexes within the sequence.

The temporal INR network represents the intra-scan motion (figure 4). The input was a frame index, and the outputs were nine PC weightings/coefficients at the queried frame to compose the frame-specific DVFs. The temporal INR shared a similar network architecture as the spatial INR, consisting of multiresolution hash encoding and nine parallel MLPs. Each MLP had one input layer, one output layer, and two hidden layers with rectified linear unit activation functions. The same set of hyper-parameters of the spatial INR (table 1) was used in the temporal INR. The feature numbers of the input and hidden layers were 32, and the feature number of the output layer was 1. Before feeding into the hash encoding, the frame index was scaled between −1 and 1. In addition, the scaled frame indexes were randomly perturbed within their frame intervals (i.e. the temporal resolution of the cine-MRI) with a Gaussian noise to force the temporal INR to learn continuous representations of PC coefficients as a function of the scaled frame index (Reed *et al* 2021).

#### 2.3.3. The progressive training scheme and loss functions

Owing to the interplay between the reference-frame reconstruction (spatial INR training), the motion solving (temporal INR training), and the limited k-space data in each frame, training the spatial and temporal INRs simultaneously from scratch was found slow and challenging. To address this challenge, we initialized the spatial INR prior to the joint training (i.e. a warm start for the joint training), and designed a STINR-MR training scheme with progressively added complexity to avoid the local minimum (Zhang *et al* 2023). The training scheme contained three stages with designated loss functions. In the first two stages, only the spatial INR was trained (without the motion solving) to construct an approximated reference frame, and the joint spatial and temporal INR training was introduced later in stage 3. To generate an initial reference-frame image, the cine-MR acquisitions ${\left\{{{\boldsymbol{w}}}_{t}({\boldsymbol{k}})\right\}}_{t=0}^{{N}_{t}-1}$ were first sorted into 10 respiratory bins, and the subset corresponding to the end-of-exhale bin, ${{\boldsymbol{w}}}_{\mathrm{exhale}}({\boldsymbol{k}}),$ was selected to reconstruct an approximated reference frame ${{\boldsymbol{z}}}_{\mathrm{exhale}}({\boldsymbol{x}})$ by non-uniform fast Fourier transform (NUFFT) (Muckley *et al* 2020). During stage 1, the spatial INR was learned directly from the NUFFT-reconstructed reference-frame MR image, by minimizing the mean absolute squared loss in the image domain:

\begin{eqnarray*}{L}_{\mathrm{image}}=\displaystyle \frac{1}{{N}_{\mathrm{voxel}}}\displaystyle \sum _{{\boldsymbol{x}}}{\left|{{\boldsymbol{z}}}_{\mathrm{ref}}\left({\boldsymbol{x}}\right)-{{\boldsymbol{z}}}_{\mathrm{exhale}}({\boldsymbol{x}})\right|}^{2},\end{eqnarray*}

where ${N}_{\mathrm{voxel}}$ represents the number of voxels of the reconstructed reference frame ${{\boldsymbol{z}}}_{\mathrm{ref}}\left({\boldsymbol{x}}\right).$ The loss function equation (5) is the sole loss function used in stage 1.

In stage 2, the similarity loss was assessed in k-space by directly using the sorted raw data from the end-of-exhale bin ${{\boldsymbol{w}}}_{\mathrm{exhale}}({\boldsymbol{k}}).$ Stage 2 aimed to mitigate the under-sampling artifacts resulting from the NUFFT reconstruction (see figure S-2 in supplementary materials). Specifically, the k-space similarity loss was formulated as the mean absolute squared error between the estimated k-space data of the reference image and the raw k-space data at the end-of-exhale bin:

\begin{eqnarray*}{L}_{k-\mathrm{space}}=\displaystyle \frac{1}{{N}_{{\boldsymbol{k}}}^{\mathrm{exhale}}}\displaystyle \sum _{{\boldsymbol{k}}}{\left|F{{\boldsymbol{[}}{\boldsymbol{z}}}_{\mathrm{ref}}]({\boldsymbol{k}})-{{\boldsymbol{w}}}_{\mathrm{exhale}}({\boldsymbol{k}})\right|}^{2},\end{eqnarray*}

where ${N}_{{\boldsymbol{k}}}^{\mathrm{exhale}}$ represents the number of k-space sampling points corresponding to the end-of-exhale bin, and *F* is the operator combining the coil sensitivity map and the Fourier transform matrix. In addition to the k-space similarity loss, stage 2 introduced a total variation (TV) loss to regularize the reference frame reconstruction (Rudin *et al* 1992):

\begin{eqnarray*}{L}_{{TV}}=\displaystyle \frac{1}{{N}_{\mathrm{voxel}}}\displaystyle \sum _{{\boldsymbol{x}}}\left|{\mathrm{\nabla }}\left|{{\boldsymbol{z}}}_{\mathrm{ref}}({\boldsymbol{x}})\right|\right|\end{eqnarray*}

The total loss function in stage 2 was a weighted sum of the similarity and regularization losses:

\begin{eqnarray*}{L}_{\mathrm{tot}}={L}_{k-\mathrm{space}}+{\lambda }_{\mathrm{TV}}\,{L}_{\mathrm{TV}}\end{eqnarray*}

The TV weighting factor ${\lambda }_{\mathrm{TV}}$ was determined by empirical searching (see section II of supplementary materials for details). The numerical values of ${\lambda }_{\mathrm{TV}}$ for different datasets were given in section 2.4.3.

For stage 3, both the spatial INR and the temporal INR were activated for learning, as shown in figure 1. The k-space similarity loss was evaluated as:

\begin{eqnarray*}{L}_{k-\mathrm{space}}^{{\prime} }=\displaystyle \frac{1}{{N}_{\mathrm{batch}}}\displaystyle \sum _{t}\displaystyle \frac{1}{{N}_{{\boldsymbol{k}}}^{t}}\displaystyle \sum _{{\boldsymbol{k}}}{\left|F{{\boldsymbol{[}}{\boldsymbol{z}}}_{t}]({\boldsymbol{k}})-{{\boldsymbol{w}}}_{t}({\boldsymbol{k}})\right|}^{2},\end{eqnarray*}

where ${N}_{\mathrm{batch}}$ represents the batch size, ${N}_{{\boldsymbol{k}}}^{t}$ denotes the number of k-space sampling points of the frame *t*. Instead of traversing all available k-space spokes, we used a subset (batch) of randomly selected k-space spokes to accelerate the computational speed and reduce the memory footprint, a strategy similar to the stochastic gradient descent algorithm. Similarly to stage 2, the TV regularization loss was used in stage 3, and the total loss function was a weighted sum of the k-space loss and the TV loss. The same weighting factors ${\lambda }_{\mathrm{TV}}$ as those in stage 2 were used.

### 2.4. Data curation and evaluation schemes

STINR-MR was evaluated using the XCAT digital phantom (Segars *et al* 2010) and two datasets of human subjects. The XCAT phantom can simulate various respiratory motion with ‘ground-truth’ images to allow quantitative evaluations and analyses. Therefore, the XCAT study served to demonstrate the feasibility and accuracy of the proposed framework as a proof-of-concept. The human subject study served to further demonstrate the applicability of the STINR-MR on real-world data. Because of the distinct nature of the datasets, we separately described them in the following subsections.

#### 2.4.1. The XCAT phantom study

We simulated 3D cine-MR images of XCAT using different respiratory motion to evaluate STINR-MR. To simulate the cine-MR images, we first used XCAT to generate a 4D-MRI set of 10 respiratory phases (from 0% to 90% with a 5 s cycle and 20 mm diaphragm peak-to-peak motion), computed inter-phase DVFs (relative to the end-of-exhale bin (0%)) via Elastix (Klein *et al* 2010), and derived principal motion components of the inter-phase DVFs via PCA. Different motion scenarios were then simulated by rescaling the principal motion components to generate intra-scan DVFs according to different motion curves and then using these DVFs to deform the end-of-exhale MRI volume to 3D cine-MRI series. The end-of-exhale XCAT MRI volume covered the whole thorax and the upper portion of the abdomen. A spherical lung tumor of 30 mm diameter was inserted into the lower lobe of the right lung, serving as a target for assessing the accuracy of solved motion. The volume size was 100 × 100 × 100 with an isotropic 4 mm resolution. Since the XCAT phantom generated magnitude-only MR images (i.e. real-valued MR images normalized in the range of [0, 1]), complex-valued images were simulated by adding spatial phase modulation to the real-valued, end-of-exhale MR image volume. The spatial phase modulation was simulated as a superposition of four sinusoidal oscillations (Zhu *et al* 2018, Terpstra *et al* 2020). The wave number of each sinusoid was randomly selected between [0.0033 mm^−1^, 0.02 mm^−1^] with a random orientation and a random phase shift. After superposing the four sinusoidal oscillations, the amplitude was normalized between 0 and 2*π*. The normalized phase map was used as the exponent to generate complex-valued phase modulation. For each simulated motion scenario, we applied the same phase modulation to the end-of-exhale MR image volume, and deformed the volume to complex-valued 3D cine-MR images via the simulated, scenario-specific intra-scan DVFs.

STINR-MR was evaluated for different motion/anatomical scenarios including: (i) various types of regular/irregular respiratory motion; and (ii) inter-scan anatomical variations between the original 4D-MRI and the cine MR scan. For (i), six types of respiration motion with various degrees of complexity were simulated. Table 2 highlights the characteristics of the motion scenarios, and figure S-1 in supplementary materials shows the corresponding center-of-mass motion trajectories of the lung tumor along the superior-inferior (SI) direction. All motion trajectories correspond to a 180 s scan and 1826 (*N*_*t*_) cine frames (each frame having a temporal resolution of 98.6 ms). Specifically, S1 was the simplest motion scenario, having small variations of the breathing amplitude along a constant baseline. On the basis of S1, S2 added a 7 mm downward baseline shift at around 90 s into the scan. S3 contained both amplitude variations and baseline shifts. S4 had a change in the breathing period and the amplitude starting from 60 s into the scan. S5 included a slow breathing motion with gradually decreasing motion amplitudes. S6 was the most complex scenario involving combined variations of breathing period, amplitude, and baseline. For (ii), we simulated inter-scan anatomical variations by reducing the lung tumor size of the end-of-exhale MR volume (from 30 to 15 mm in diameter), before mapping it to 3D cine-MR images using the intra-scan DVFs of the motion scenario S1. Due to space limitations, the results of the inter-scan anatomical variation test were presented in supplementary materials (section V.3).

**Table 2.** Summary of motion characteristics of six motion scenarios in the XCAT phantom study.

Motion scenario	Motion characteristics
S1	Amplitude variations
S2	Baseline shifts
S3	Amplitude variations and baseline shifts
S4	Respiratory period/amplitude variations
S5	Slow breathing with a small amplitude variation
S6	Combination of respiratory period/amplitude variations and baseline shifts

From the simulated ‘ground-truth’ complex-valued 3D cine-MR images, we generated the k-space data assuming free-breathing MR acquisitions, for STINR-MR reconstruction and evaluation. For simplicity, we considered the MR acquisitions to involve a single coil with a uniform sensitivity map covering the whole field-of-view. We used the gradient echo-based pulse sequences, with the k-space data acquisition simulated via 3D golden-mean Koosh-ball trajectories (Winkelmann *et al* 2007, Chan *et al* 2009, Feng 2022). The Koosh-ball trajectory was non-Cartesian and comprised of readout lines in the radial directions (i.e. spokes). Each readout line passed through the k-space origin, with its orientation order following the golden-mean algorithm (Chan *et al* 2009). Via the Koosh-ball trajectory, the central region of k-space was oversampled to be more motion robust. The data can be easily sorted by motion for self-navigation, which renders the Koosh-ball trajectory particularly suitable for dynamic 3D-cine MRI (Lingala *et al* 2016, Stemkens *et al* 2018). Although the Koosh-ball trajectory was the focus of this study, STINR-MR can be readily applied to other 3D trajectories (e.g. Liao *et al* 1997, Burdumy *et al* 2017), as the image reconstruction and motion solving were irrelevant to the k-space trajectory specifics.

For k-space simulation, we used a TR = 5.8 ms (Deng *et al* 2016), corresponding to 17 spokes per frame (each frame has a 98.6 ms temporal resolution). The number of radial spokes for a fully-sampled 3D scan is ∼24 674 spokes (estimated by assuming a uniform sampling in the polar and azimuthal angles of the radial spokes), and the corresponding under-sampling ratio is ∼1451. In our evaluation, we also tested using even fewer spokes per frame to reconstruct more frames and further increase the temporal resolution to evaluate the relationship between the reconstruction accuracy and the under-sampling ratio, and to investigate how high the temporal resolution can be. In detail, we used 4, 8, or 17 spokes to represent a frame for STINR-MR reconstruction, which corresponded to 23.2 ms, 46.4 ms, and 98.6 ms in temporal resolution, for the S1 motion scenario study. Due to space limitations, the results of the varying temporal resolution tests were presented in supplementary materials (section V.4).

STINR-MR requires a PCA-based motion model as input (figure 1), which can come from two sources: (1) the PCA model from the originally-simulated 4D-MRI (as described above), which in clinical practice can be a previously-acquired 4D-MRI that offers offline information (offline PCA); and (2) the PCA model directly derived from 4D-MRIs reconstructed using the online cine MR acquisitions (online PCA). In cases where a previously-acquired 4D-MRI may not be available, we can sort the k-space data of cine MR acquisitions into 10 bins to reconstruct an online 4D-MRI via NUFFT, and perform PCA without relying on any prior data. In this study, we evaluated both approaches and compared their accuracy.

We evaluated the accuracy of the reconstructed 3D cine-MR images and the accuracy of the tumor motion solved by intra-scan DVFs, by comparing them with the simulated ‘ground-truth’. The reconstructed reference-frame MR images were visually examined, and the whole sequence of 3D cine-MR images was quantitatively evaluated using the relative error (RE) metric:

\begin{eqnarray*}{\mathrm{RE}}=\sqrt{\frac{\displaystyle {\sum }_{{\boldsymbol{x}}\,}{\left(\left|{{\boldsymbol{z}}}_{t}({\boldsymbol{x}})\right|-\left|{{\boldsymbol{z}}}_{t}^{{gt}}({\boldsymbol{x}})\right|\right)}^{2}}{\displaystyle {\sum }_{{\boldsymbol{x}}}{\left|{{\boldsymbol{z}}}_{t}^{{gt}}({\boldsymbol{x}})\right|}^{2}}},\end{eqnarray*}

where ${{\boldsymbol{z}}}_{t}^{{gt}}$ denotes the ‘ground-truth’ image. The accuracy of the tracked tumor motion by solved intra-scan DVFs was evaluated using the tumor center-of-mass error (COME) and the Dice similarity coefficient (DSC). The COME measures the center-of-mass distance between the DVF-propagated tumor location and the ‘ground-truth’ tumor location. The DSC is defined by

\begin{eqnarray*}{\mathrm{DSC}}=\frac{2\times \left|Y\cap {Y}^{{gt}}\right|}{\left|Y\right|+\left|{Y}^{{gt}}\right|},\end{eqnarray*}

where *Y* and *Y* ^*gt*^ denote the DVF-propagated and the ‘ground-truth’ tumor contours, respectively.

#### 2.4.2. The human subject study

In addition to the XCAT simulation study, STINR-MR was also evaluated on two MRI datasets of human subjects. The first dataset was from the University of Texas Southwestern Medical Center (UTSW), consisting of free-breathing scans of five patients with respiration-induced liver motion. Each patient had a 4D THRIVE scan and a 3D T2-weighted scan, and both scans covered the abdominal region. Table 3 summarizes the MR acquisition and reconstruction parameters. Due to the fast imaging sequence, 4D THRIVE exhibited lower image quality and contrast. On the other hand, the 3D T2-weighted image, acquired with gating, showed better image quality and a higher signal-to-noise ratio. Based on the two data sets, a 4D T2-weighted MRI set was generated, using a DL-based framework (dual-supervised deformation estimation model) (Xiao *et al* 2022). From the 4D T2-weighted MRI set, a PCA-based motion model was derived for each patient for 3D cine-MRI simulation.

**Table 3.** Summary of MR acquisition and reconstruction parameters of the University of Texas Southwestern Medical Center (UTSW) dataset.

	4D THRIVE MRI	3D T2-weighted MRI
Scanner	Philips ingenia ambition	Philips ingenia ambition
Magnetic field strength (T)	1.5	1.5
Sequence	4D THRIVE fat saturated	SPIR MultiVane-XD
Flip angle (degree)	10	90
Repetition time (ms)	3.49	5,227
Echo time (ms)	1.57	70
Volume size (width × height × slice)	480 × 480 × 53	512 × 512 × 53
Voxel size (mm^3^)	0.875 × 0.875 × 3	0.879 × 0.879 × 3

All MR images in the UTSW dataset were post-processed, magnitude-only images, thus the raw k-space data were inaccessible. Accordingly, we simulated the complex-valued MR data by adding spatial phase modulations to the magnitude-only images (Zhu *et al* 2018, Terpstra *et al* 2020). 3D cine-MR images of a slow breathing pattern and an irregular breathing pattern, as well as the k-space acquisitions, were simulated similarly to the XCAT study. Before adding the phase modulations, the MR images were resampled to a size of 128 × 128 × 48 with a spatial resolution of 3 × 3 × 3 mm^3^. The total scan time was simulated as 3 min. The k-space sampling used the 3D golden-mean Koosh-ball radial trajectory with TR = 4.4 ms to match the MR-MOTUS liver subject acquisition scenario (Huttinga *et al* 2021). Each frame consisted of 22 radial spokes, corresponding to a temporal resolution of 96.8 ms and an under-sampling ratio of ~689. There were 128 readout points per radial spoke. Based on the simulated ‘ground-truth’ cine-MR images, the image quality and motion tracking accuracy of STINR-MR were evaluated using the image relative error metric and COME/DSC of the reconstructed dynamic liver volumes.

The second dataset contained a free-breathing scan of a healthy human subject acquired by a 1.5 T MRI scanner (Ingenia, Philips Healthcare) from the University of Medical Center Utrecht (UMCU) (Huttinga *et al* 2021). For the k-space acquisition, the phase array consisted of 12 anterior and 12 posterior receive coils, and the sensitivity map and the noise covariance matrix were provided for each coil. The pulse sequence was a steady-state spoiled gradient echo sequence. The TR and echo time were 4.4 ms and 1.8 ms, respectively, and the flip angle was 20°. The k-space was acquired via a 3D golden-mean Koosh-ball radial trajectory. The total scan duration was 297.4 s, resulting in 67 280 radial spokes with 232 readout points per spoke. The first 900 spokes were discarded to allow the system to reach a steady state. The scan covered the thoracic and abdominal regions.

Different from the XCAT simulation study, there is no prior 4D-MRI available for the UMCU dataset to build the PCA motion model. Thus, we built an online PCA model directly using the available k-space data. In detail, we extracted a surrogate signal representing the respiratory motion from the k-space signals (Huttinga *et al* 2022). The k-space signals of each coil at the origin $\left\{{{\boldsymbol{w}}}_{t}({\boldsymbol{k}}=0)\right\}$ were extracted from all sequential radial readouts, and consolidated as a 24-channel time series. It was subsequently processed by a low-pass filter using the Kaiser window method (Kaiser and Schafer 1980) to remove high-frequency noises. PCA was then performed on the filtered time series, and the principal component with the largest spectral density in the frequency range between 0.1 and 0.5 Hz (corresponding to the respiratory motion frequency range) was selected as the surrogate signal. Based on the surrogate signal, the radial spokes were sorted into 10 respiratory phases and reconstructed into a 4D-MRI using NUFFT (Muckley *et al* 2020). The reconstructed image size was 150 × 150 × 150, with a 3.0 × 3.0 × 3.0 mm^3^ resolution. From the 4D-MRI, a PCA-based motion model was generated, as described in section 2.2.

For the UMCU study, the L2 similarity loss (equation (5)) of STINR-MR was defined for each of the 24 coils and then summed together. To achieve a balance between the noise suppression and the temporal resolution, we binned 68 radial spokes per coil into a frame, which corresponds to a temporal resolution of 299.2 ms and an under-sampling ratio of ~816. Since no ‘ground-truth’ was available for the human study, STINR-MR’s performance was assessed by visual inspection and quality evaluation of the reconstructed reference-frame MR image. For quality evaluation, we measured the sharpness of the reconstructed reference frame using a variance-based metric (Ferzli and Karam 2005). The variance metric was calculated as the mean variance of the whole reference-frame MR image (Ferzli and Karam 2005). A higher value indicates a sharper image with less motion blurriness. We also compared the liver center-of-mass motion tracked by STINR-MR with the k-space surrogate’s motion. The liver center-of-mass was calculated by contouring the liver in the reference frame and then propagating the contour by the intra-scan DVFs solved by STINR-MR.

#### 2.4.3. Other hyper-parameters of the STINR-MR framework and the training details

The Adam optimizer was used for STINR-MR training. Under the progressive training scheme (section 2.3.3), the learning rate of the MLPs in the spatial INR was reset at the beginning of each stage. For the XCAT study and the UTSW study, we used learning rates of 1 × 10^−3^, 2 × 10^−5^, and 2 × 10^−6^ empirically for the first, second, and last stages, respectively. For the UMCU healthy subject dataset, we used learning rates of 1 × 10^−3^, 1 × 10^−5^, and 1 × 10^−7^ for the three stages, respectively. The reduction of the learning rate between the first and the second stages was to account for the substantial increase of the similarity loss, when switching from the image domain (equation (5)) to the k-space domain (equation (6)). Similarly, the learning rate was reduced at the beginning of stage 3 to account for the loss function increase caused by the introduction of motion dynamics. For the XCAT study and the UTSW study, the first, second, and last stages were trained by 500, 1500, and 1000 epochs, respectively. For the UMCU study, the corresponding epochs were 500, 300, and 1100, respectively. For the joint training of the third stage of all studies, one epoch contained 60 frames (${N}_{\mathrm{batch}}$ of equation(9)) randomly selected from the MR acquisitions, which was determined to balance the training speed/stability and to avoid the temporal aliasing, while being bounded by the available memory in the graphic processing unit (GPU) (NVIDIA A100). The weighting factors ${\lambda }_{\mathrm{TV}}$ of the XCAT, the UTSW dataset, and the UMCU dataset were empirically set to 7 × 10^−5^, 7 × 10^−5^, and 2 × 10^−6^, respectively. The overall training time was ~20 min for the XCAT and UTSW studies, and ∼100 min for the UMCU study, respectively. The training time difference was mainly due to the size differences of the reference frame, the k-space complexity (single-channel vs. multi-channel), and the underlying complexity of the reconstructed anatomy. The GPU memory consumption was measured and reported in the Results section.

#### 2.4.4. The comparison study with MR-MOTUS

STINR-MR was compared with MR-MOTUS (Huttinga *et al* 2020, Huttinga *et al* 2021), a model-based and non-rigid motion estimation method that was recently developed. MR-MOTUS had three features distinct from STINR-MR: (i) the model was formulated for k-space data of a single channel, so multi-coil data had to be compressed into a single virtual channel prior to the MR-MOTUS reconstruction. (ii) The reference-frame MR image was from a previously-acquired MRI, or reconstructed from the motion-sorted k-space data, with no additional refinement during the motion estimation stage. (iii) MR-MOTUS used a low-rank motion model to regularize the motion estimation and B-spline-based parametrization of the spatial and temporal motion components for dimension reduction.

MR-MOTUS was evaluated on all three datasets (XCAT, UTSW, and UMCU). Due to large computational resource demands, the reference-frame MR images were down-sampled by a factor of 2 by MR-MOTUS (which was also used in the original implementation (Huttinga *et al* 2021)). Moreover, the whole MR data sequence was partitioned into smaller batches, and the cine-MR images were independently reconstructed from each batch. Due to space limitations, details of MR-MOTUS implementation can be found in supplementary materials (section IV.1). Nonparametric Wilcoxon signed-rank tests of image quality and tumor localization accuracy between MR-MOTUS and STINR-MR (offline and online PCA) were performed to evaluate the significance levels of observed differences.

#### 2.4.5. The comparison study with a deep learning-based method (TEMPEST)

In addition to the comparison with MR-MOTUS, STINR-MR was compared with a DL-based MRI reconstruction technique TEMPEST (Terpstra *et al* 2021), which is considered as a state-of-the-art method for 3D cine-MRI reconstruction. TEMPEST reconstructs a 3D MR image by estimating a 3D DVF respective to a fully-sampled static MR image, via a three-level multiresolution deformable registration approach. The network took an under-sampled dynamic MRI and a prior static MRI as two channels of inputs and estimated a 3D DVF respective to the static image. The multi-resolution motion estimation doubled the spatial resolution at each higher level to improve the motion estimation accuracy. The 3D cine-MR images can be reconstructed by sequentially inputting the dynamic images of the cine acquisition into TEMPEST. In this study, we adopted the same network architecture and hyperparameters as those used by Terpstra *et al* (2021).

The TEMPEST model was separately trained on the XCAT simulation and UTSW patient datasets, as both datasets involved different anatomical sites and image features. Due to space limitations, details of the training scheme can be found in supplementary materials (section IV.2). After the network training, the XCAT-based TEMPEST model was evaluated on the six motion scenarios (S1–S6) of the XCAT phantom using 17 spokes (i.e. 98.6 ms temporal resolution). Similarly, the patient-based TEMPEST model was evaluated on the slow and irregular motion scenarios of the UTSW dataset, using 22 spokes per frame (i.e. 96.8 ms temporal resolution). Wilcoxon signed-rank tests of the image quality and tumor localization accuracy between TEMPEST and STINR-MR (offline and online PCA) were performed to evaluate the significance levels of observed differences.

## 3. Results

### 3.1. The XCAT phantom study

**Figure 5.**
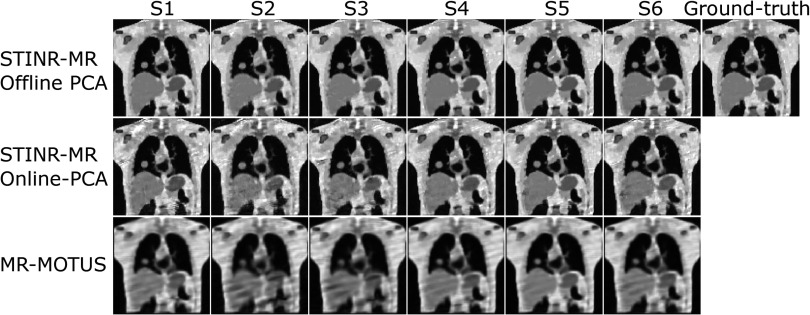
Comparison of reconstructed reference-frame MR images for the six motion scenarios (S1–S6) of the XCAT study using three methods. Each column (columns 1–6) shows the reconstructed reference-frame images of one motion scenario, and column 7 shows the reference image of the XCAT simulation.

**Figure 6.**
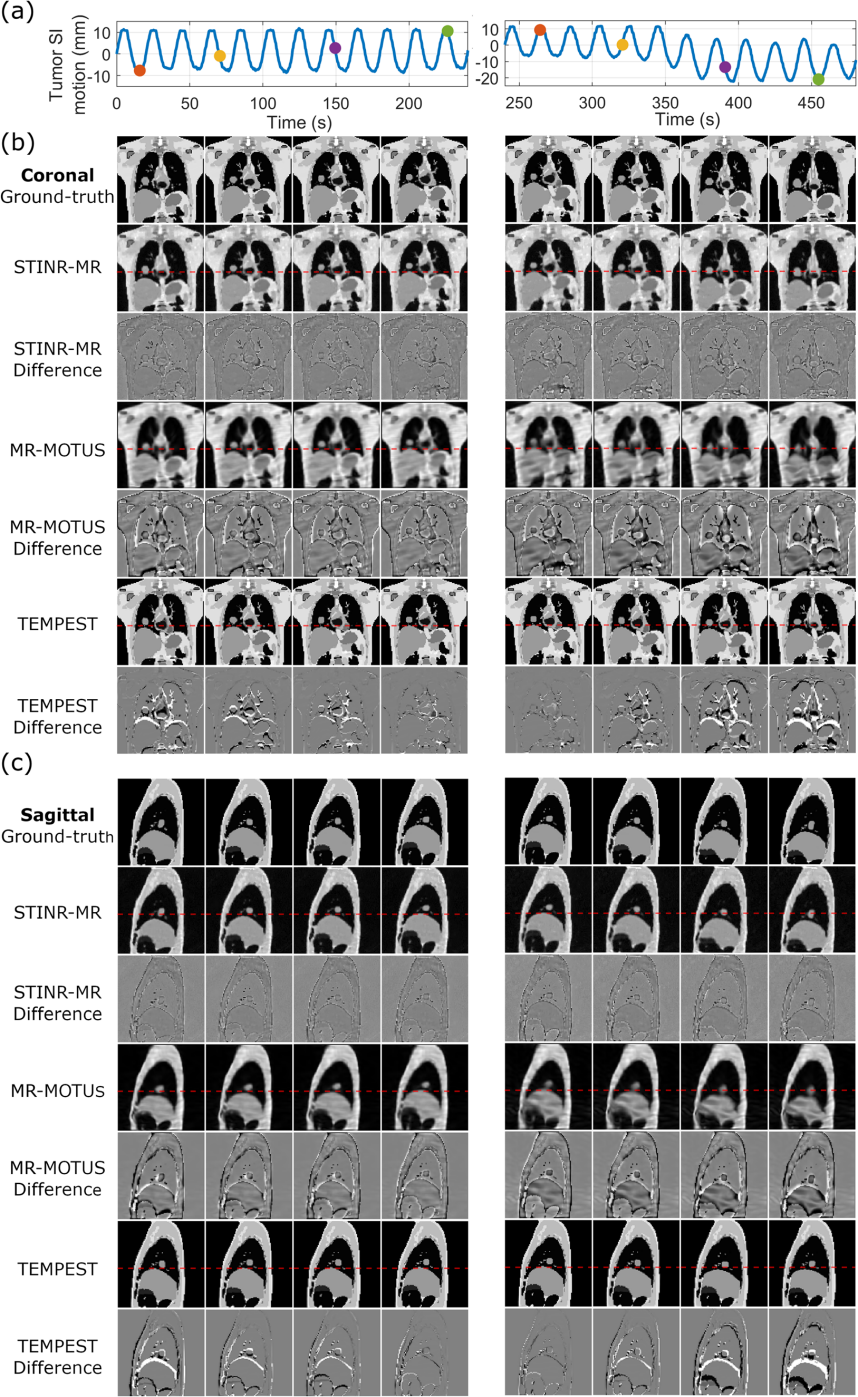
Comparison between reconstructed cine-MR images by STINR-MR (offline PCA), MR-MOTUS, and TEMPEST for the XCAT S1 (left column) and S2 (right column) motion scenarios in the (b)coronal and (c) sagittal views. The corresponding difference images between the ‘ground-truth’ and the reconstructions were also shown. The top panels (a) in each view depict the STINR-MR-solved lung tumor motion trajectories in the SI direction, and the dots indicate the motion states selected for plotting.

Figure 5 visually compares reconstructed reference-frame MR images by three methods (STINR-MR: offline PCA; STINR-MR: online PCA; and MR-MOTUS) for the XCAT study. Note that TEMPEST used the same reference frame for all motion scenarios, which was the end-of-exhale bin of the simulated prior 4D-MRI (i.e. the Reference image on the top-right corner). The GPU memory consumption for the XCAT study was 18.8 GB for STINR-MR. STINR-MR with offline PCA presented images with the highest quality for all motion scenarios, while the reference-frame images of MR-MOTUS showed strip artifacts due to under-sampling and motion. STINR-MR with online PCA presented images with overall good quality, while some artifacts can be observed due to the inaccuracy of the online-derived PCA models (due to irregular and non-periodic motion, intra-phase motion, and sorting errors). Table 4 summarizes the mean relative error metric averaged over the entire 3D cine-MR image series. All Wilcoxon signed-rank tests between STINR-MR (offline or online PCA) and the other methods yielded *p* values less than 10^−3^, demonstrating statistical significance. Results of the cine-MR reconstruction under a lower spatial resolution were presented in supplementary materials (section V.1). Figure 6 compares the ‘ground-truth’ and the reconstructed cine-MR images of selected cine frames, for the S1 and S2 motion scenarios of the XCAT phantom study. Similarly, STINR-MR showed the best overall image quality and motion accuracy. MR-MOTUS presented more blurred cine images, resulting from the under-sampled reference-frame image reconstruction after motion sorting. Compared with MR-MOTUS, STINR-MR employed all available k-space data for joint image reconstruction and motion estimation, achieving superior quality in the reference-frame MRI and the correspondingly solved intra-scan motion. In comparison, TEMPEST had relatively worse performance in motion estimation, and the error increased when the motion (relative to the reference image) increased.

**Table 4.** Mean ± SD relative errors of reconstructed 3D cine-MR images over the whole motion sequence for the various motion scenario study of XCAT.

Motion scenario	STINR-MR offline PCA	STINR-MR online PCA	MR-MOTUS	TEMPEST
S1	**0.159 ± 0.002**	0.223 ± 0.006	0.279 ± 0.007	0.228 ± 0.052
S2	**0.172 ± 0.004**	0.249 ± 0.010	0.304 ± 0.009	0.268 ± 0.069
S3	**0.162 ± 0.004**	0.235 ± 0.017	0.296 ± 0.006	0.235 ± 0.062
S4	**0.162 ± 0.003**	0.205 ± 0.007	0.274 ± 0.005	0.214 ± 0.050
S5	**0.158 ± 0.002**	0.207 ± 0.007	0.273 ± 0.008	0.224 ± 0.052
S6	**0.165 ± 0.002**	0.219 ± 0.005	0.280 ± 0.008	0.233 ± 0.050

Table 5 summarizes the lung tumor localization accuracy measured over the whole sequences of 3D cine-MR images. Both variants of STINR-MR outperformed MR-MOTUS and TEMPEST and achieved sub-voxel localization accuracy. All the Wilcoxon signed-rank tests between STINR-MR (offline or online PCA) and the other methods yielded *p* values less than 10^−3^, demonstrating statistical significance. A comparison of the tumor center-of-mass motion curves in the SI direction as a function of time was given in supplementary materials due to the space limits (figure S-4). Whereas the DL-based TEMPEST method demonstrated comparable mean relative errors to MR-MOTUS (and both were inferior to the two STINR-MR variants), the motion tracking results of TEMPEST were much worse than the other methods. In addition, the performance of TEMPEST was unstable, with standard deviations much larger than the other three methods. We also tested TEMPEST using a substantially higher sampling ratio (272 spokes per cine frame) on the S1 motion scenario, and obtained a lung tumor COME of 2.3 ± 1.3 mm, which is close to the reported localization accuracy (Terpstra *et al* 2021). These results showed that TEMPEST had difficulty extracting useful image features for motion estimation under extremely under-sampled scenarios.

**Table 5.** Lung tumor localization accuracy for the six motion scenarios of the XCAT study, measured by the tumor center-of-mass error (COME) and the Dice similarity score (DSC). The values were presented in terms of mean and standard deviation.

Motion scenario	COME (mm)				DSC			
	STINR-MR Offline PCA	STINR-MR Online PCA	MR-MOTUS	TEMPEST	STINR-MR Offline PCA	STINR-MR Online PCA	MR-MOTUS	TEMPEST
S1	**1.0 ± 0.5**	1.4 ± 0.7	3.5 ± 0.9	5.3 ± 4.1	**0.92 ± 0.02**	0.90 ± 0.03	0.80 ± 0.04	0.77 ± 0.16
S2	**0.9 ± 0.4**	2.5 ± 1.1	3.1 ± 1.0	9.0 ± 7.1	**0.91 ± 0.02**	0.86 ± 0.05	0.73 ± 0.05	0.65 ± 0.23
S3	**1.0 ± 0.5**	2.1 ± 1.4	3.2 ± 1.0	6.5 ± 5.0	**0.92 ± 0.03**	0.86 ± 0.05	0.78 ± 0.04	0.72 ± 0.17
S4	**0.6 ± 0.3**	1.3 ± 0.5	3.2 ± 1.0	5.2 ± 3.4	**0.93 ± 0.01**	0.89 ± 0.02	0.81 ± 0.05	0.77 ± 0.14
S5	**0.9 ± 0.4**	1.3 ± 0.5	3.5 ± 1.0	5.1 ± 4.0	**0.92 ± 0.02**	0.88 ± 0.02	0.80 ± 0.05	0.79 ± 0.16
S6	**1.0 ± 0.4**	1.4 ± 0.8	4.0 ± 1.1	5.6 ± 4.1	**0.92 ± 0.02**	0.90 ± 0.03	0.78 ± 0.05	0.76 ± 0.16

### 3.2. The human subject study

**Figure 7.**
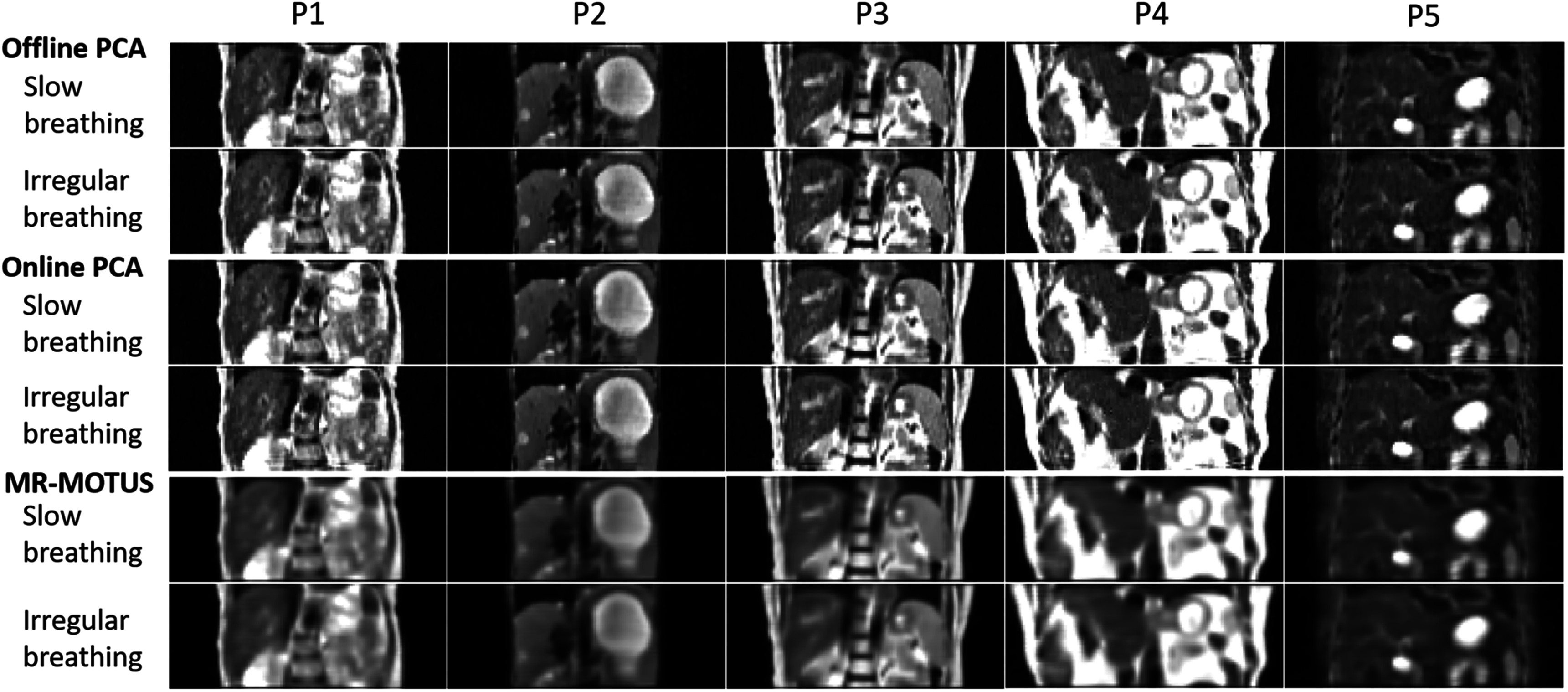
Comparison of reconstructed reference-frame MR images by STINR-MR (offline and online PCA) and MR-MOTUS for the slow and irregular breathing scenarios of the five patients (P1–P5) in the UTSW dataset.

**Figure 8.**
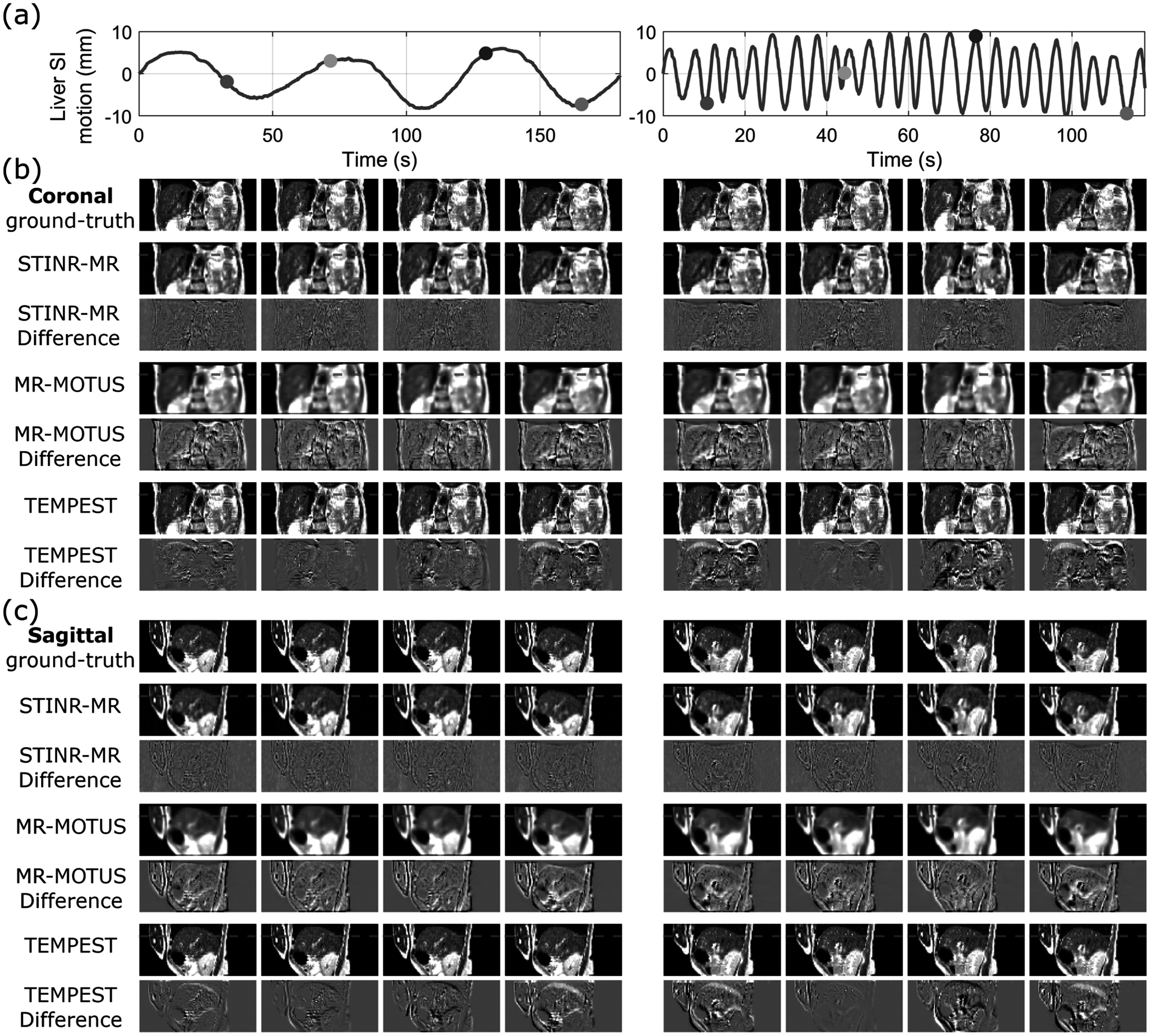
Comparison of reconstructed cine-MR images using STINR-MR (offline PCA), MR-MOTUS, and TEMPEST, with the ‘ground-truth’ images in the (b) coronal and (c) sagittal views for the slow (left column) and irregular (right column) breathing scenarios of the UTSW patient (P1) simulation study. The corresponding difference images were also shown. The top panels (a) present the STINR-MR-solved liver motion trajectories in the SI direction, and the dots indicate the motion states selected for plotting.

Figure 7 presents a visual comparison between the reconstructed reference-frame images, for the slow and irregular motion scenarios of the five liver patients (P1–P5) in the UTSW dataset, and table 6 summarizes the relative errors of the reconstructed 3D cine-MR images of both breathing scenarios. Overall, STINR-MR demonstrated superior performance in the mean relative error compared to MR-MOTUS and TEMPEST. In contrast to the XCAT study, the image qualities of the offline- and online-PCA variants were more comparable in the UTSW patient study. Compared with STINR-MR, MR-MOTUS displayed lower image quality, as its reference frames were reconstructed from under-sampled reconstructions after motion-sorting. TEMPEST used the same reference-frame MRI for both slow and irregular motion scenarios, which was the end-of-exhale phase of the prior 4D-MRI. TEMPEST exhibited the lowest relative error for the P3’s slow breathing scenario. It is partly due to its use of an ‘ideal’ reference-frame image for motion estimation, which is generally not achievable in real clinical settings. For the relative error metric, all Wilcoxon signed-rank tests between STINR-MR (offline and online PCA) and MR-MOTUS or TEMPEST yielded *p* values <10^−3^. The GPU memory consumption of STINR-MR was 15.0 GB for the UTSW patient dataset simulation study. Figure 8 compares the ‘ground-truth’ and the STINR-MR (offline PCA) reconstructed cine-MR images of selected cine frames for the slow and irregular breathing scenarios of P1. Table 7 shows the liver motion tracking accuracy of the five patients in the UTSW dataset, in terms of the liver COME and DSC. All Wilcoxon signed-rank tests of COMEs and DSCs between STINR-MR (offline and online PCA) and MR-MOTUS or TEMPEST yielded p values <10^−3^, except for the test of COME between offline-PCA STINR-MR and MR-MOTUS of P2’s irregular breathing scenario (*p* = 0.010). Due to the space limits, a comparison between tracked liver center-of-mass motion curves by different methods and the ‘ground-truth’ in the SI direction was given in supplementary materials (figure S-7).

**Table 6.** Mean ± SD relative errors of reconstructed 3D cine-MR images over the whole motion sequence for the five patients in the UTSW dataset.

Patient ID	Slow breathing				Irregular breathing			
	STINR-MR offline PCA	STINR-MR online PCA	MR-MOTUS	TEMPEST	STINR-MR Offline PCA	STINR-MR Online PCA	MR-MOTUS	TEMPEST
P1	**0.161 ± 0.004**	0.167 ± 0.004	0.442 ± 0.003	0.168 ± 0.063	0.161 ± 0.005	**0.160** ± **0.004**	0.437 ± 0.007	0.197 ± 0.076
P2	0.180 ± 0.011	**0.169** ± **0.006**	0.317 ± 0.029	0.205 ± 0.070	0.161 ± 0.011	**0.161** ± **0.007**	0.321 ± 0.031	0.207 ± 0.074
P3	0.139 ± 0.005	0.139 ± 0.003	0.302 ± 0.009	**0.102** ± **0.038**	**0.122** ± **0.005**	0.129 ± 0.003	0.308 ± 0.016	0.123 ± 0.049
P4	**0.128** ± **0.003**	0.129 ± 0.003	0.294 ± 0.033	0.187 ± 0.072	0.118 ± 0.004	**0.118** ± **0.003**	0.293 ± 0.034	0.187 ± 0.074
P5	**0.151** ± **0.006**	0.155 ± 0.005	0.331 ± 0.040	0.216 ± 0.085	**0.145** ± **0.006**	0.148 ± 0.007	0.341 ± 0.043	0.225 ± 0.092

**Table 7.** Liver motion tracking accuracy of the five patients in the UTSW dataset. The results are presented as mean ± SD.

Patient ID	COME (mm)							
	Slow breathing				Irregular breathing			
	STINR-MR offline PCA	STINR-MR online PCA	MR-MOTUS	TEMPEST	STINR-MR offline PCA	STINR-MR online PCA	MR-MOTUS	TEMPEST
P1	1.6 ± 0.3	**1.2** ± **0.3**	2.1 ± 0.6	3.3 ± 2.0	**1.6** ± **0.5**	1.8 ± 0.4	2.1 ± 0.8	4.2 ± 2.6
P2	**1.0** ± **0.2**	1.4 ± 0.2	2.0 ± 1.0	1.8 ± 1.0	2.0 ± 0.2	**1.4** ± **0.2**	2.2 ± 1.2	1.8 ± 1.1
P3	**1.0** ± **0.3**	1.2 ± 0.3	3.4 ± 2.3	3.5 ± 2.0	2.1 ± 0.4	**1.6** ± **0.3**	3.0 ± 1.8	4.3 ± 2.4
P4	**1.1** ± **0.3**	1.2 ± 0.3	2.4 ± 2.5	4.4 ± 3.2	**0.8** ± **0.4**	1.1 ± 0.2	1.9 ± 1.4	4.4 ± 3.3
P5	**0.5** ± **0.2**	1.3 ± 0.2	2.7 ± 2.2	3.8 ± 2.7	**0.5** ± **0.2**	1.1 ± 0.2	2.4 ± 1.8	4.0 ± 2.9

Patient ID	DSC							
	
	Slow breathing				Irregular breathing			
		
	STINR-MR offline PCA	STINR-MR online PCA	MR-MOTUS	TEMPEST	STINR-MR offline PCA	STINR-MR online PCA	MR-MOTUS	TEMPEST

P1	**0.96** ± **0.01**	**0.96** ± **0.01**	0.86 ± 0.02	0.94 ± 0.03	**0.96** ± **0.01**	0.95 ± 0.01	0.87 ± 0.01	0.93 ± 0.04
P2	**0.96** ± **0.01**	**0.96** ± **0.01**	0.94 ± 0.01	0.96 ± 0.02	**0.97** ± **0.01**	0.96 ± 0.01	0.94 ± 0.01	0.96 ± 0.02
P3	**0.97** ± **0.01**	0.96 ± 0.01	0.93 ± 0.02	0.95 ± 0.03	**0.97** ± **0.01**	0.93 ± 0.01	0.93 ± 0.02	0.94 ± 0.03
P4	**0.96** ± **0.01**	0.95 ± 0.01	0.92 ± 0.04	0.91 ± 0.06	**0.97** ± **0.01**	0.96 ± 0.01	0.92 ± 0.03	0.91 ± 0.06
P5	**0.97** ± **0.01**	0.96 ± 0.01	0.93 ± 0.03	0.94 ± 0.04	**0.97** ± **0.01**	0.96 ± 0.01	0.93 ± 0.03	0.94 ± 0.04

**Figure 9.**
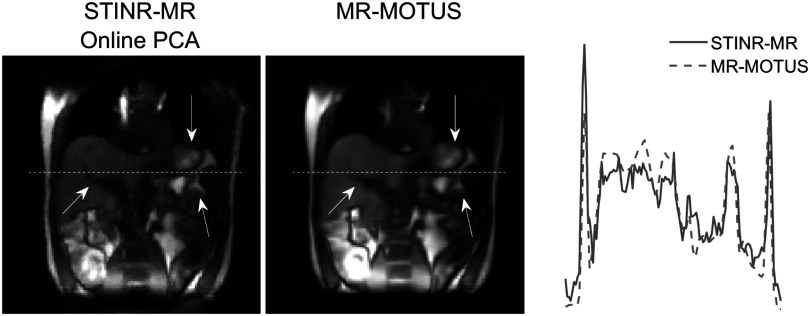
Comparison of reconstructed reference-frame MR images and the corresponding line profiles for the UMCU human subject study. The corresponding location of the line profiles was indicated by the horizontal dashed lines, and the arrows highlighted the over-smoothed regions of MR-MOTUS.

Figure 9 compares the reference-frame MR images reconstructed by STINR-MR (via online PCA) and by MR-MOTUS for the UMCU study, using clinically acquired k-space data. The GPU memory consumption of STINR-MR was 66.2 GB. A line profile across the liver was also compared. The STINR-MR reference frame showed sharper organ boundaries, while the MR-MOTUS reference frame appeared overly smoothed with blurriness potentially caused by intra-scan motion. The calculated variance metrics for STINR-MR and MR-MOTUS were 0.0079 and 0.0073, respectively. It shows that STINR-MR provided sharper reference-frame MR images after joint reconstruction and deformable registration, which yielded more anatomical details to guide the diagnosis and/or treatments.

**Figure 10.**
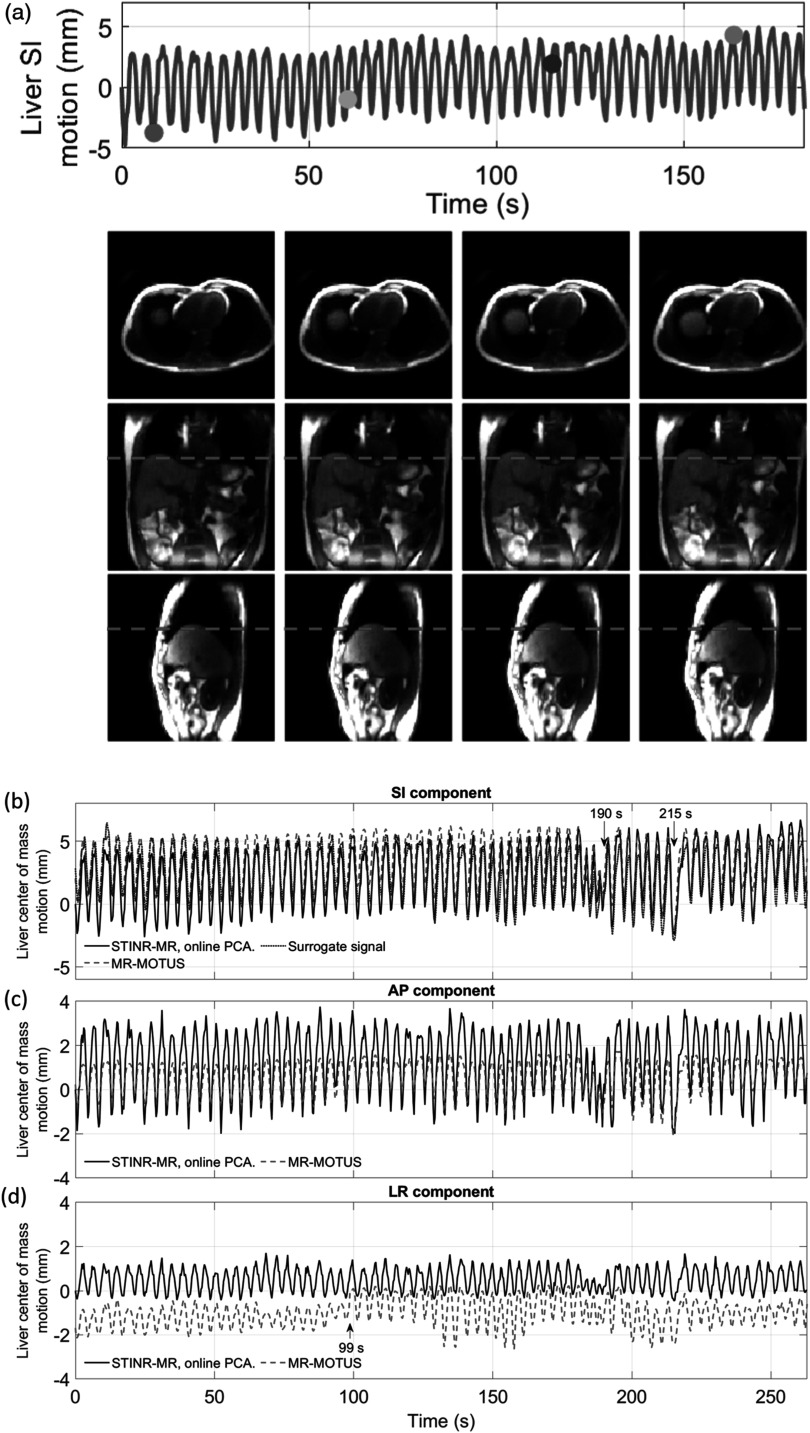
(a) Selected 3D cine MRI frames reconstructed by STINR-MR in three views for the UMCU human subject study. The top panel presents the STINR-MR-solved liver motion curve, and the dots indicate the motion states selected for plotting. (b)–(d) Comparison of the liver center-of-mass motion estimated by STINR-MR and MR-MOTUS. Subfigures 10(b)–(d) show the SI, AP, and LR components of the liver center-of-mass motion, respectively. For comparison, the surrogate signal extracted from the k-space origin was plotted in subfigure (b).

Figure 10(a) presents selected 3D cine MRI frames reconstructed by STINR-MR in three views. Figures 10(b)–(d) compare the tracked liver center-of-mass motion in the SI, anterior-posterior (AP), and left-right (LR) directions, by STINR-MR and MR-MOTUS. For comparison, the surrogate signal directly extracted from the k-space was plotted in figure 10(b) (section 2.4.2). The surrogate signal was extracted from the origin of the k-space data via denoising and principal component analysis to show the general motion trend, and may not fully represent the detailed liver motion. Along the SI direction, the amplitude variations at around 190 s and the breathing period variations at around 215 s of the surrogate signal were reproduced by both STINR-MR and MR-MOTUS. Overall, STINR-MR and MR-MOTUS solved similar motion curves in the SI direction, but STINR-MR had slightly larger SI motion amplitudes than MR-MOTUS. In addition, MR-MOTUS solved smaller AP motion amplitudes than STINR-MR, and it also had a relative baseline shift in the LR direction. The smaller motion amplitudes of MR-MOTUS could be due to the motion blurriness and over-smoothing observed in its reference-frame MR image (figure 9). Considering that the spatial resolution of the MR-MOTUS reconstruction was 6.7 × 6.7 × 6.7 mm^3^, the 2 mm relative shift in the LR direction could be due to a sub-voxel reconstruction offset. For MR-MOTUS, general amplitude variations/discontinuities in the LR direction were also observed at several temporal sections, especially from around 99 s. Such variations are likely due to the batch-based reconstruction of MR-MOTUS to address the memory limits (each batch has around 33 s of data), which yielded slightly different low-rank DVF bases across batches that might affect the cross-batch motion amplitude consistency.

## 4. Discussion

In this study, we proposed a joint reconstruction and deformable registration framework, STINR-MR, for 3D cine-MRI reconstruction. STINR-MR used a spatial INR and a temporal INR, together with a PCA-based motion model, to reconstruct 3D cine MR images with superior spatial and temporal resolutions. STINR-MR decoupled the challenging spatiotemporal inverse problem into the joint training of two INR networks to separately capture the spatial information and the temporal motion, which allows high-quality dynamic image reconstruction from significantly under-sampled data via a ‘one-shot’ learning scheme. The introduction of the PCA-based motion model helps to regularize the motion fields and reduce the corresponding solution space, and allows the STINR-MR framework to capture highly irregular motion patterns (figures 6 and 8, tables 4–7, and supplementary file: figures S-4 and S-7).

Due to the inherent limitations of MR acquisition, there are fundamental tradeoffs between temporal resolution, spatial resolution, and signal-to-noise ratio. Many reconstruction algorithms used compressed sensing that exploits spatial and temporal sparsity of cine acquisitions (e.g. Lustig *et al* 2006) or incorporated motion estimation and compensation to facilitate the reconstruction (e.g. Jung *et al* 2009). However, due to the still-limited MR acquisition speed and lengthy reconstruction time, the majority of cine MRI applications are limited to 2D. These studies typically reported a maximum in-plane resolution of ~1.2 × 1.2 mm^2^ with a relatively thick slice of ~8 mm and a temporal resolution equal to or worse than 30 ms (Menchon-Lara *et al* 2019). In contrast, STINR-MR can reconstruct large field-of-view, 3D cine MR volumes with 3 × 3 × 3 mm^3^ spatial resolution and ~100 ms temporal resolution.

### 4.1. Performance of STINR-MR and online versus offline PCA models

STINR-MR proves robust to the simulated motion and anatomical variations (figures 5–8, tables 4–7, Figures S-4 toS-7 and Tables S-4 to S-7 in supplementary materials), for both the online and the offline PCA-based variants. Comparing online and offline PCA, the latter generally provides better results, which is expected as the offline PCA is close to the ‘perfect’ motion model as long as the underlying anatomy remains similar between the prior 4D-MRI and the new cine MR acquisitions. In contrast, the online PCA has to be built on-the-fly from the cine MR acquisitions and is susceptible to the irregularity of the online motion. Of the six motion scenarios (table 2 and supplementary materials: figure S-1), the two scenarios with substantial baseline shifts, S2 and S3, are more challenging for STINR-MR using an online PCA motion model. The relatively worse performance is expected as the baseline shifts will lead to prominent intra-phase motion artifacts in the reconstructed online 4D-MRI, and these artifacts will propagate into the corresponding online PCA motion model to reduce the accuracy of STINR-MR.

Although the offline PCA shows generally more favorable results compared to online PCA, the differences are not substantial, especially in terms of the tumor localization accuracy (tables 5 and 7, and supplementary materials: tables S-5 and S-7, and figure S-4). For the inter-scan tumor shrinkage scenario (table S-5), the offline PCA performs slightly worse than the online PCA, as the underlying anatomical change impacts the validity of the offline PCA motion model that is built from prior offline 4D-MR images. In addition, in this study, we did not consider and simulate artifacts in the offline 4D-MRI, which can be caused by under-sampling and motion irregularities in real clinical practices. The artifacts in the offline 4D-MRI will similarly propagate into the offline PCA motion model, as for the case of online 4D-MRI, and impact the results of the STINR-MR based on the offline PCA model. We expect the performance gap to be even smaller between online and offline PCA-based models when such scenarios are considered, and future comprehensive studies using more real patient data are warranted.

### 4.2. Simulation and human studies

In this study, we used XCAT simulations to evaluate STINR-MR. One challenge of the XCAT study is to simulate complex-valued MR signals with spatial phase modulation. We adopted a phase modulation simulation strategy used in previous deep learning-based works (Zhu *et al* 2018, Terpstra *et al* 2020), which shows good generalizability toward real clinical data. Various phase maps were simulated based on the superposition of four sinusoidal oscillations to generate complex-valued MR images with meaningful real and imaginary parts. However, such simulations may not fully represent the phase maps in clinical data. The reference-frame MR images reconstructed in the XCAT study (figure 5) appear to have more strip artifacts than those reconstructed in the human subject study (figures 7 and 9). These artifacts were caused by two factors: the uncertainty of the online PCA motion model and the phase modulation simulated. The online PCA motion model was derived from the 4D-MRI reconstructed from the online cine-MR signals. The motion-sorting for the 4D-MRI reconstruction may introduce intra-phase motion artifacts and under-sampling artifacts, especially for highly irregular motion curves. Correspondingly, the accuracy of the principal motion components, which are derived from inter-phase DVFs of the 4D-MRI, will be degraded and the errors will propagate into the 3D cine-MRI reconstruction process. When the errors of the PCA motion model were coupled with a simulated phase map which is less smooth, the reconstruction becomes more challenging and leads to the observed strip artifacts in some of the XCAT reconstructions (STINR: online PCA). In addition to the XCAT study, we used two human subject datasets to further evaluate STINR-MR. No such artifacts were observed for the STINR-MR (online-PCA) in the UTSW patient study (figure 7) and the healthy human subject study (figure 9). However, the inaccurate online PCA motion model remains a limitation of the current STINR-MR framework. A solution to this challenge is to develop a prior motion model-free framework that directly learns and optimizes the motion model during the cine-MRI reconstruction. Currently, we are investigating such a data-driven motion model for STINR-MR, which is beyond the scope of the current work and will be reported in a future study.

The UMCU healthy human subject’s results show that the motion solved by STINR-MR is similar to that of MR-MOTUS, and to motion surrogate signals directly tracked from the k-space. The UMCU study further validates the clinical applicability of STINR-MR, while future investigations are warranted to quantitatively compare STINR-MR to other methods using real patient data to evaluate their accuracy and efficiency. To provide a ‘gold-standard’ reference to evaluate STINR-MR, we can acquire self-navigation motion surrogate signals interlaced into the pulse sequence, while such evaluation is currently limited to 1D (Huttinga *et al* 2022). For 3D evaluation, anthropomorphic and motion-enabled MR phantoms can be used with well-controlled, ‘known’ motion. Such a phantom is currently under development in our group for future studies (Chiu *et al* 2022).

### 4.3. GPU memory consumption

Since the spatial INR and the temporal INRs are compact and lightweight, the dynamic MR sequences can be represented with a small GPU memory footprint. However, during the loss function computation of each iteration, if we choose to infer the spatial and temporal INRs into cine-MRIs of all temporal frames, it will lead to a long computation time and increase the memory load. Thus, in consideration of efficiency and GPU memory consumption, we randomly selected 60 temporal frames out of the whole sequence during each step to compute the loss and update the networks. The GPU memory consumptions were 18.8 GB, 15.0 GB, and 66.2 GB for the XCAT phantom study, the UTSW patient study, and the UMCU healthy subject study, respectively. For the healthy subject study, the higher GPU memory consumption was mainly due to the size of reconstructed images (i.e., 150 × 150 × 150 versus 100 × 100 × 100 for XCAT). The increased GPU memory consumption can be a bottleneck of STINR-MR when high-resolution reconstruction is needed. The same memory limit and bottleneck also apply to MR-MOTUS, since both methods rely on the whole k-space dataset (rather than a few k-space spokes like TEMPEST) to reconstruct the dynamic sequence. Such bottlenecks can be addressed by quickly evolving GPU hardware with increasing memories. We can also reduce the batch size of the training to fit in the GPU limit at the potential cost of the training stability (the memory footprint was less than 30 GB when the batch size was reduced to 16). Training using multiple GPUs may also potentially address this problem, which however is beyond the scope of our study.

### 4.4. Comparison between TEMPEST and STINR-MR

Comparing TEMPEST with STINR-MR, STINR-MR is an unsupervised ‘one-shot’ learning technique that does not require any pre-training. Thus STINR-MR does not require ‘gold-standard’ DVFs, which can be challenging and costly to obtain. In addition, STINR-MR is not susceptible to potential domain shifts between training and testing. Such domain shifts can include motion pattern changes and image intensity distribution variations, which can be challenging issues for TEMPEST. Since TEMPEST relies on a high-quality prior image to serve as a reference, the acquisition condition changes for new imaging sessions may introduce additional uncertainties. In our XCAT and UTSW studies, such domain shifts and uncertainties were minimized as the same digital phantom and reference-frame images were used. However, TEMPEST still generated much worse results compared with STINR-MR, especially in terms of motion tracking (tables 5 and 7). The worse performance could be caused by the extreme under-sampling scenarios tested in our study (17/22 3D spokes for an under-sampling ratio of ∼1451/∼689), as quantitative evaluations in the original TEMPEST publication were reported for under-sampling ratios less than 50. One advantage of TEMPEST over STINR-MR, however, is that TEMPEST allows real-time imaging by generating new MRIs through extremely limited data, while STINR-MR requires access to the full k-space sequence to reconstruct all the dynamics together. Introducing additional motion prediction modules into STINR-MR may extend its utility to real-time imaging, which is currently under investigation.

### 4.5. Comparison between MR-MOTUS and STINR-MR

Compared with MR-MOTUS, a major advantage of STINR-MR is its ability to fine-tune the reference-frame MR image during the joint image reconstruction and motion solving stage, which helps to remove aliasing and motion artifacts using all k-space data and a simultaneously-optimized motion model. In contrast, MR-MOTUS reconstructed the reference frame before the motion solving, and the motion/under-sampling artifacts of the reference-frame MR image were propagated and subsequently affected the accuracy of solved intra-scan DVFs. MR-MOTUS was computationally demanding and required substantial memory footprints and computing time. As a result, in our study, the whole sequence of MR acquisitions was partitioned into batches for motion estimation to meet the memory constraint. While partitioning can accelerate the reconstruction process and reduce the memory requirements, some discontinuities were observed in the solved motion across the batches (figure 10(d)). There was currently no mechanism to enforce consistency and coherence throughout the whole sequence, as the motion estimation between different batches was independent. Such a discontinuity can be mitigated by initializing the basis DVFs of one batch with the ones solved from the previous batch, while such a strategy will cause prolonged reconstruction time (the batches cannot be parallelized), and the errors may propagate from batch to batch. In contrast, STINR-MR is a lightweight and compact framework that can reconstruct the whole sequence of 3D cine-MRI with >1000 frames in a single shot.

## 5. Conclusion

STINR-MR presents a joint image reconstruction and deformable registration framework to reconstruct 3D cine-MRI, by using powerful spatial and temporal implicit neural representations with learning-based hash encoding. The results demonstrated that STINR-MR can reconstruct dynamic volumetric MR images of >1000 frames and <100 ms temporal resolutions per frame, with superior accuracy and efficiency. With its cine-imaging capability, STINR-MR can capture irregular and aperiodic motion patterns and the underlying 3D anatomy to improve MR-guided interventions, such as MR-guided radiotherapy.

## Data Availability

The data cannot be made publicly available upon publication because they contain sensitive personal information. The data that support the findings of this study are available upon reasonable request from the authors.

## Ethical statement

The UTSW dataset was retrospectively collected from an approved study at UT Southwestern Medical Center on 31 August, 2023, under an umbrella IRB protocol 082013–008 (Improving radiation treatment quality and safety by retrospective data analysis). This is a retrospective analysis study and not a clinical trial. No clinical trial ID number is available. Individual patient consent was signed for the anonymized use of the imaging and treatment planning data for retrospective analysis. The study was conducted in accordance with the principles embodied in the Declaration of Helsinki.
